# The Effect of Serotonin Receptor 5-HT1B on Lateral Inhibition between Spiny Projection Neurons in the Mouse Striatum

**DOI:** 10.1523/JNEUROSCI.1037-20.2021

**Published:** 2021-09-15

**Authors:** Stefan Pommer, Yumiko Akamine, Serge N. Schiffmann, Alban de Kerchove d'Exaerde, Jeffery R. Wickens

**Affiliations:** ^1^Neurobiology Research Unit, Okinawa Institute of Science and Technology Graduate University, Onna-son, Okinawa 904-0495, Japan; ^2^Laboratory of Neurophysiology, Université Libre de Bruxelles Neuroscience Institute, Université Libre de Bruxelles, Brussels 1070, Belgium

**Keywords:** GABA, lateral inhibition, MSN, serotonin, SPN, synapse

## Abstract

The principal neurons of the striatum, the spiny projection neurons (SPNs), make inhibitory synaptic connections with each other via collaterals of their main axon, forming a local lateral inhibition network. Serotonin, acting via the 5-HT1B receptor, modulates neurotransmitter release from SPN terminals in striatal output nuclei, but the role of 5-HT1B receptors in lateral inhibition among SPNs in the striatum is unknown. Here, we report the effects of 5-HT1B receptor activation on lateral inhibition in the mouse striatum. Whole-cell recordings were made from SPNs in acute brain slices of either sex, while optogenetically activating presynaptic SPNs or fast-spiking interneurons (FSIs). Activation of 5-HT1B receptors significantly reduced the amplitude of IPSCs evoked by optical stimulation of both direct and indirect pathway SPNs. This reduction was blocked by application of a 5-HT1B receptor antagonist. Activation of 5-HT1B receptors did not reduce the amplitude of IPSCs evoked from FSIs. These results suggest a new role for serotonin as a modulator of lateral inhibition among striatal SPNs. The 5-HT1B receptor may, therefore, be a suitable target for future behavioral experiments investigating the currently unknown role of lateral inhibition in the function of the striatum.

**SIGNIFICANCE STATEMENT** We show that stimulation of serotonin receptors reduces the efficacy of lateral inhibition between spiny projection neurons (SPNs), one of the biggest GABAergic sources in the striatum, by activation of the serotonin 5-HT1B receptor. The striatum receives serotonergic input from the dorsal raphe nuclei and is important in behavioral brain functions like learning and action selection. Our findings suggest a new role for serotonin in modulating the dynamics of neural interactions in the striatum, which extends current knowledge of the mechanisms of the behavioral effects of serotonin.

## Introduction

The basal ganglia play an important role in integrative brain functions like motor control or learning, and are involved in neurologic disorders such as Parkinson's disease and Huntington's chorea. The striatum is the main input nucleus of the basal ganglia. Spiny projection neurons (SPNs; Wilson and Groves, 1980; Somogyi et al., 1981) constitute ∼95% of all striatal neurons (Kemp and Powell, 1971; Oorschot, 1996). In addition to producing a main axon that projects to the output nuclei of the striatum SPNs also give rise to local axon collaterals (Wilson and Groves, 1980). The synaptic boutons of the local axon collaterals synapse on other SPNs (Somogyi et al., 1981; Wilson, 1994; Oorschot et al., 2002) forming a lateral inhibition-type neural network. The functional inhibitory interactions between SPNs are considered weak because of their sparse connectivity and high failure rates (Czubayko and Plenz, 2002; Tunstall et al., 2002; Koos et al., 2004; Tepper and Bolam, 2004; Bolam et al., 2006). The behavioral significance of lateral inhibition in the striatum is unknown, in part because there has been no way to selectively block it. In this study, we investigated the effect of serotonin 5-HT1B receptors on lateral inhibition in the striatum.

The striatum receives serotonin innervation from the raphe nuclei in a spatially graded manner, with highest concentrations toward the ventral striatum as well as in the globus pallidus (GP) and substantia nigra (Steinbusch, 1981; Di Matteo et al., 2008; Pan et al., 2010). There are no intrinsic serotonergic cells in the striatum but there is a range of serotonin receptors present (Miguelez et al., 2014). These receptors are involved in serotonin modulation of corticostriatal (Mathur et al., 2011) and thalamostriatal (Cavaccini et al., 2018) glutamatergic input via presynaptic and postsynaptic mechanisms. The serotonin receptor subtype 5-HT1B is expressed by SPNs (Maroteaux et al., 1992; Boschert et al., 1994; Langlois et al., 1995; Pauwels, 1997; Sari et al., 1999; Sari, 2004; Mengod et al., 2006). This G-protein-coupled receptor acts via G_i_ α subunits leading to a decrease of cellular cAMP and also activates the MAPK pathway (Masson et al., 2012; Wang et al., 2013). In addition, several groups linked the receptor to ionic potassium and calcium channels (Mizutani et al., 2006; Heblinski et al., 2019). In the substantia nigra and GP 5-HT1B receptors of SPNs are expressed in presynaptic terminals (Maroteaux et al., 1992; Boschert et al., 1994; Sari et al., 1999; Jolimay et al., 2000; Hoyer et al., 2002). There, the 5-HT1B receptor acts as a heteroreceptor, regulating the release of GABA, allowing control of basal ganglia motor responses (Johnson et al., 1992; Stanford and Lacey, 1996; Ghavami et al., 1999; Riad et al., 2000; Sari, 2004). Conceivably, 5-HT1B receptors play a similar role in the terminals of the local axon collaterals of SPNs in the striatum. However, there have been no previous studies on the effect of serotonin or 5-HT1B receptor on GABA release in the striatum.

In the dorsal striatum, spiny neurons fall in two groups: SPNs expressing dopamine D1 receptors and projecting to the substantia nigra pars reticulata (SNr); and SPNs expressing D2 receptors and projecting to the GP (Ince et al., 1997; Bolam et al., 2000, 2006; Burke et al., 2017). Differences in lateral inhibition between D1 and D2-SPNs have been reported (Taverna et al., 2008; Planert et al., 2010). However, there is currently no evidence for differential expression of 5-HT1B receptors on D1 and D2-SPNs.

We hypothesize that 5-HT1B receptors modulate lateral inhibition in the dorsal striatum. To test this hypothesis, we measured the effect of these receptors on lateral inhibition using whole-cell recording *in vitro*. An optogenetic approach was used to selectively activate D1 or D2-SPNs. We measured the effects of pharmacological manipulation of 5-HT1B receptors on IPSCs in channelrhodopsin-negative neurons. We found that lateral inhibition was decreased by 5-HT1B receptor activation in both D1→D2 and D2→D1 connections.

## Materials and Methods

### Animals

All animal procedures were conducted in accordance with the Animal Experiment Regulations of the Okinawa Institute of Science and Technology Graduate University and were approved by the Animal Care and Use Committee (protocol 2016–134). Male and female mice of the following strains were used: Tg(Drd1-Cre)FK150Gsat/Mmucd (Gensat), Adenosine A2A receptor-Cre BAC transgenic mice (Alban de Kerchove d'Exaerde) as described previously (Durieux et al., 2009, 2012) and B6;129P2-*Pvalb^tm1(cre)Arbr^*/J (JAX stock #017320). The transgenic mice were backcrossed to C57BL/6J >5 generations (PV-Cre) and 12 generations (Drd1-Cre and A2A-Cre), before use.

### Surgery and viral injections

All surgeries were done using a stereotaxic system (Leica Angle Two). The animals were anesthetized with isoflurane (IsoFlo) for the surgery. The skin was opened before determining striatal injection sites with the stereotaxic system. A small opening was made in the skull above the target location at bregma 0.86 mm, medial-lateral (ML) −1.53 mm (Franklin and Paxinos, 2013). Subsequently, the left hemisphere was injected with 300 nl viral solution at dorsal-ventral (DV) −3 mm. After that, the wound was closed and sutured before the animal was left for recovery. The virus rAAV5/EF1a-DIO-hChR2(H134R)-eYFP (UNC Vector Core) was used to express Channelrhodopsin2 (ChR2) for optogenetic stimulation experiments. Serotype 5 provided high neuronal and low transynaptic infection (Zincarelli et al., 2008; Aschauer et al., 2013). The ChR2(H134R) version was selected because previous studies have shown successful expression and excitation in the same mice strains (Lopez-Huerta et al., 2016).

### Immunofluorescence

For characterization and colocalization of 5-HT1B with neuronal markers, animals were perfused with 40-ml 4% formaldehyde in 1× PBS (10010023, Invitrogen). Brains were extracted and further fixed for 3 d in 4% formaldehyde at 4°C. Coronal slices (60 µm) were made with a vibratome (VT1000S, Leica). Brain slices were blocked in 1× PBS with 5% goat serum and 0.2% saponin for 1 h at room temperature before incubated with primary antibodies and 0.02% saponin for 48 h at 4°C. Then, slices were rinsed with 1× PBS and incubated with secondary antibodies conjugated to fluorophores for 4 h at room temperature before mounting. The following antibodies were used: 5-HT1B (ASR-022, Alomone Labs, 1:200), choline acetyltransferase (AB144P, Chemicon, 1:150), and DARPP-32 (611520, BD Biosciences, 1:250). Fluorophore-conjugated secondary antibodies were matched according to the primary antibody host and minimized emission spectrum overlap from Alexa Fluor 568 (1:500), Alexa Fluor 594 (1:500), and Alexa Fluor 647 (1:500) from ThermoFisher Scientific. Cell nuclei were counterstained with DAPI (Nuc Blue Fixed Cell Stain Ready Probes reagent, Invitrogen).

Imaging data were acquired using confocal and Airyscan microscopy (LSM780/880, Carl Zeiss). Subsequent analysis was performed with Fiji (Schindelin et al., 2012; Rueden et al., 2017) and Imaris (Bitplane). All images are maximum intensity projections of the full slice Z-Stacks or regions of interest. Coronal brain slices were reconstructed with the mouse brain atlas by Paxinos and Franklin (Franklin and Paxinos, 2013).

#### Pharmacology/drug treatment

The following drugs were added by perfusion during *in vitro* recordings: 2 µm CP-93129 (1032, TOCRIS), 10 µm SB-216641 (1242, TOCRIS), 0.5 µm tetrodotoxin (TTX; 206-11071, Wako Chemicals), 10 µm bicuculline (2503, TOCRIS), and 2 mm kynurenic acid (KA; K3375, Sigma-Aldrich). KA was used to block potential excitatory transmission through AMPA, NMDA, and kainate receptors. Drugs showed an initial effect after 2–3 min.

#### Acute slice preparation

The animals were anesthetized with IsoFlo before decapitation. The brain was removed and put into ice-cold NMDG cutting solution containing 93 mm NMDG, 2.5 mm KCl, 10 mm MgCl2, 0.5 mm CaCl2, 30 mm NaHCO3, 25 mm glucose, 5 mm sodium ascorbate, 2 mm thiourea, 3 mm sodium pyruvate, 20 mm HEPES, 1.2 mm NaH_2_PO_4_, oxygenated with 95% O_2_ + 5% CO_2_. Slices containing the striatum were cut at a 45° angle to horizontal plan to preserve striatal fiber bundles and corticostriatal connections (Wickens and Arbuthnott, 2010). The slices (250 µm thick) were cut using a vibratome (VT1000S, Leica) and mounted on a porous membrane before transferred to artificial CSF (ACSF) containing 125 mm NaCl, 2.5 mm KCl, 1.25 mm NaH_2_PO_4_, 1 mm MgCl2, 2 mm CaCl2, 25 mm NaHCO3, 15 mm glucose, saturated with 95% O_2_ and 5% CO_2_. The slices were incubated for half an hour at 35°C before being left to recover for at least 30 min at room temperature.

### Slice electrophysiology

**Figure 1.**
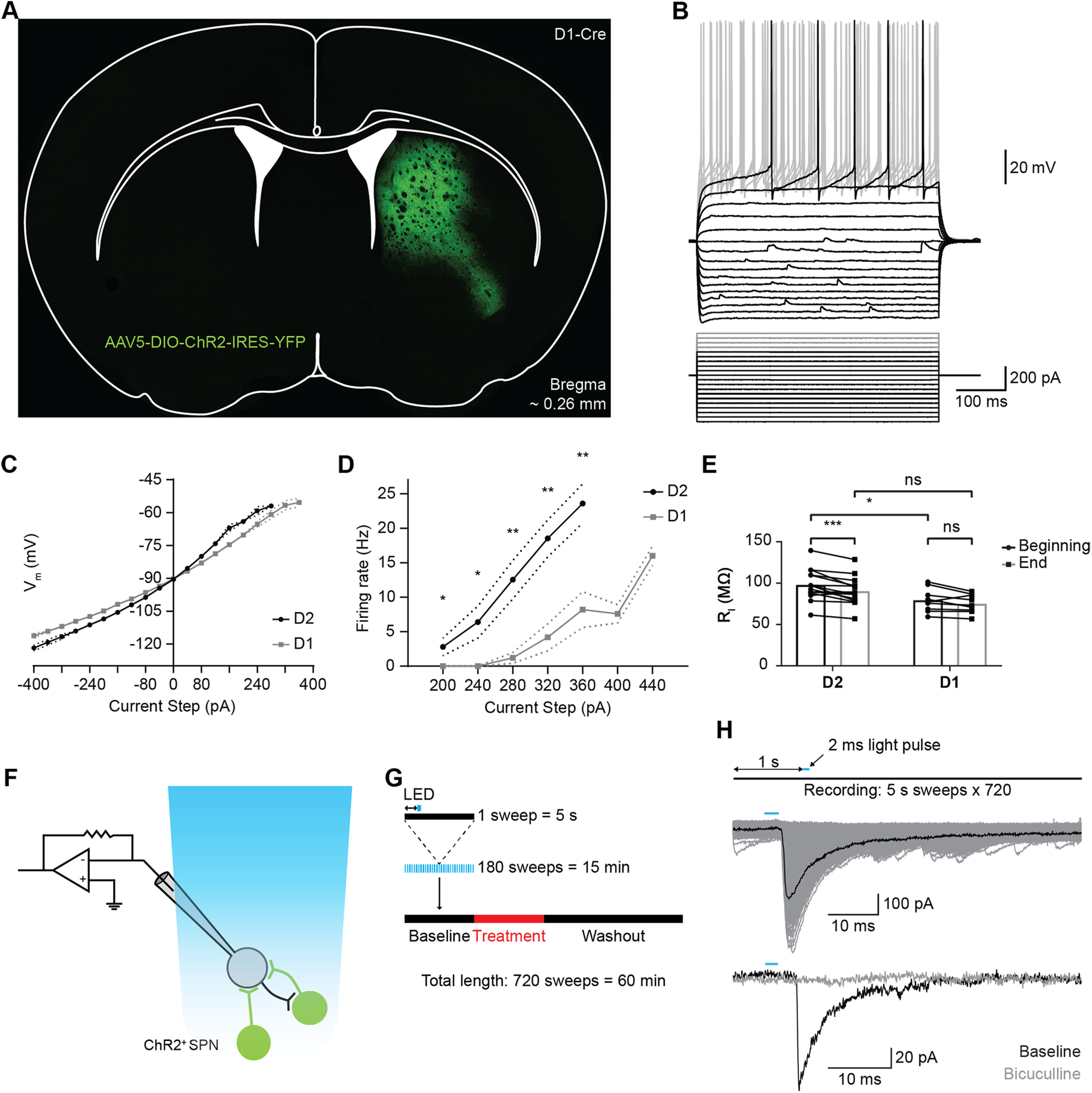
Properties of postsynaptic ChR2- neurons in ChR2 injected mice. ***A***, Specific expression of ChR2 in a D1-Cre mouse. Position: bregma ∼0.26 mm. ***B***, Superimposed voltage traces of an example SPN in response to a series of current steps shows inward rectification. Small depolarizations in the membrane potential are spontaneous events. Only the first AP-eliciting step is shown in black for clarity. The cell was held at −90 mV. ***C***, Average IV-curve of subthreshold membrane potential from D1 and D2-SPNs. The voltage response curve is close to linear for hyperpolarizing steps and increases exponentially above −90 mV. V**alues are mean ± SEM. Dotted lines represent SEM were applicable. *N* = 14/10, D2/D1. ***D***, Average firing frequency versus current intensity for D1 and D2-SPNs. Values are mean ± SEM. Dotted lines represent SEM were applicable. *N* = 14/9, D2/D1. ***E***, Average input resistance (R_I_) of postsynaptic SPNs at the beginning (first minute) and end (last minute) of recording. The input resistance is slightly lower for D1-SPNs. ***F***, Whole-cell patch clamp of SPNs recording IPSCs evoked by optogenetic stimulation of lateral ChR2^+^ SPNs. ***G***, Recording protocols for ChR2-evoked IPSCs in voltage clamp configuration. Membrane current is recorded in repeated 5-s sweeps. A single 2-ms light pulse (blue bar) is given one second (double arrowheads) after the start of each sweep. ***H***, Example IPSC response (middle graph) to presynaptic optogenetic stimulation (upper graph). APs were triggered with a 2-ms light pulse. Gray traces are an overlay of repeated stimulation. Lower graph, Example trace of an IPSC after a 2-ms light pulse in control ACSF (black trace) and in the presence of bicuculline (gray trace). All recordings were done in the presence of KA. ns, *p* > 0.05; *p < 0.05, **p < 0.01, ***p < 0.001, ****p < 0.0001.

Whole cell recordings were made from SPNs in the dorsal striatum identified by size, electrophysiological properties and/or expression of eYFP (Olympus BX51WI). Borosilicate glass pipettes (4–9 MΩ) were pulled on a horizontal electrode puller (P-97, Sutter Instruments) and filled with a high chloride internal solution containing 30 mm KH_2_PO_4_, 100 mm KCl, 10 mm NaCl, 2 mm MgCl_2_, 0.5 mm EGTA, 10 mm HEPES, 2 mm ATP, and 0.03 mm GTP, adjusted to pH 7.4 with KOH. This shifted the equilibrium potential for Cl^–^ ions to −3 mV, amplifying Cl^–^ currents. It also made Cl^–^ currents depolarizing toward the threshold potential. Slice recordings were made in a chamber perfused by ACSF + KA at 30°C at a flow rate of 3 ml/min. Acquisition was done using pCLAMP 10.2, Digidata 1440A and a MultiClamp700B amplifier (Molecular Devices). The data were digitized at 20 kHz, filtered with a 4-kHz Bessel filter and analyzed further with Clampfit 10.2 (Molecular devices), Axograph X 1.54 (John Clements) and Matlab (MathWorks) as indicated. Optogenetic stimulation of SPNs expressing ChR2 (Fig. 1*F*) was performed using blue (470 nm) LED illumination through the objective (LEX2-B, BrainVision/SciMedia). One hundred percent intensity at the focal plane was 17.95 mW/mm^2^. This was calculated by measuring the light power at focal point through the objective with a photometer (Q8230, ADVANTEST) to 542 µW and dividing it over the illuminated area of 0.0302 mm^2^. The diameter of the focal spot was estimated from the length of the diagonal of the camera field of view. Light pulses (2- or 5-ms duration) were transmitted via a neutral-density filter (25% attenuation, U-25ND25, Olympus) to produce a final intensity of 4.49 mW/mm^2^. Input resistance was measured using steps of 20 mV (voltage clamp) or 20 pA (current clamp) during recording.

### Recording protocol

Action potentials (APs) were optically evoked at 5-s intervals over 60 min of recording (Fig. 1*G*). After a 15-min baseline, drugs (serotonin receptor agonist CP-93129 or TTX) were applied for 15 min. In drug control experiments, the serotonin receptor antagonist SB-216641 was applied for 45 min from the start of the recording to block the effects of CP-93129. No-drug control experiments were also performed.

### Data analysis

SPNs with access resistance above 22 MΩ at the start or end of the recording were rejected. SPNs with >20% input resistance change over the course of the recording or sudden changes of resistance were deemed unstable and discarded from further analysis. The same applied to recordings that failed to complete the baseline or treatment period because of cell death or loss of seal. IPSCs were identified using a template-matching algorithm in Axograph X (Clements and Bekkers, 1997). A two-pass approach was used to improve detection and reduce false positives. A first template was generated as average of 12 manually identified IPSCs from a 10-ms window after the light pulse. The template length was set to 5 ms with a 5-ms preceding baseline. This template was used for the first pass in the respective window of 10 ms after each light pulse. The threshold for detection was determined by manual comparison of the detected events from a typical experiment. The threshold was set to 2.5 times the standard deviation of the preceding noise. Comparison with manual detection showed the algorithm provided robust detection with a low false positive rate (≤3%). The detected events were captured and aligned. A time course average of the detected events served as basis for the second template using the same parameters. The second template was used for the second pass through the data set. Detected events from the second pass were considered true events and analyzed further. IPSCs with amplitudes of <20 pA could not be distinguished from noise and were excluded from the analysis.

### Statistical analysis

Statistical analysis was performed using Prism 8 (GraphPad). Values are reported as mean ± SD/SEM. Two-way repeated measures ANOVA was used for the analysis of the excitability difference between D1 and D2-SPNs as well as the SPN input resistance over time and between subtypes. The data were matched by subcolumn, spread across rows, and followed up by a multiple comparison test with Bonferroni–Dunn and Holm–Šídák's correction. The assumption of sphericity was rejected for the analysis of excitability and corrected with the Geisser–Greenhouse method. We compared the spike threshold between D1 and D2-SPNs with Welch's *t* test. In the analysis of effects of CP-93129 in optogenetic experiments, recordings lasting <45 min were excluded because they did not contain equal periods of baseline, drug diffusion and washout. Recordings with 2- and 5-ms light stimulation were pooled since each pulse produced a single presynaptic AP. Repeated measures one-way ANOVA with Tukey's or Holm–Šídák's multiple comparison test was used to compare the effect of 5-HT1B on lateral inhibition before, during and after application of CP-93129, SB-216641, and TTX. The assumption of sphericity was rejected and corrected with the Geisser–Greenhouse method. Additionally, multiple *t* tests were used to compare IPSCs in CP-93129 treatment and no-drug control groups. Correction for multiple comparison was done using the Holm–Šídák method. The significance level was set to ɑ = 0.05; ns, *p* > 0.05; *p < 0.05, **p < 0.01, ***p < 0.001, ****p < 0.0001.

## Results

### Optical stimulation causes GABAergic IPSCs in ChR2-negative SPNs

We tested whether the effects of 5-HT1B agonists were mediated by receptors on SPN collaterals using an optogenetic approach. Specific expression of ChR2 in D1 or D2-SPNs allowed recording of lateral inhibition in ChR2-negative SPNs. Striatal injection of DIO-ChR2(H134R)-eYFP in D1 or A2a-Cre mice led to strong expression in D1 or D2-SPNs (Fig. 1*A*).

The electrophysiological characteristics of SPNs in these two transgenic mouse lines have not been detailed previously. Patched neurons were considered to be SPNs if they exhibited electrophysiological properties typical of SPNs in wild-type mice (Gertler et al., 2008). The putative SPNs that were negative for ChR2 were used to measure these physiological properties. Voltage responses to current pulses showed inward rectification in response to hyperpolarizing current pulses (Fig. 1*B*) and delayed AP firing in response to suprathreshold current pulses (Fig. 1*B*, bottom trace). Prolonged suprathreshold stimulation evoked regular repetitive firing with no indication of spike-frequency adaptation (Fig. 1*B*, upper traces). These are typical electrophysiological properties of SPNs (Kawaguchi et al., 1989; Wilson, 1992).

To compare electrophysiological characteristics of D1 and D2-SPNs, the membrane potential and firing rate in response to current steps were measured in D2-SPNs (*N* = 15) and D1-SPNs (*N* = 10). Each cell was stimulated with at least 20 current steps (−400–360 pA). The I-V relationship was nonlinear, with decreased slope at hyperpolarized membrane potentials and increased slope at more depolarized potentials (Fig. 1*C*). One D1-SPN and one D2-SPN were excluded because of missing data of I-V responses. To examine the relationship between current intensity and firing rate, two additional steps were applied (400–440 pA) if the depolarizing steps were not sufficient to trigger APs, to reach threshold levels and SPN firing. In general, the average firing rate increased steadily with each current step (Fig. 1*D*). D2-SPNs had a higher firing rate, lower firing threshold (200 pA) and no spike frequency adaptation. In contrast, D1-SPNs required higher current steps (280 pA) and had lower firing rates than D2-SPNs. Additionally, five D1-SPNs required >360-pA depolarizing current to trigger APs, which was above the current intensity tested for other cells. This resulted in a decrease of the average firing rate at the higher current intensity. Overall, the firing rate increased less with each step in D1-SPNs compared with D2-SPNs.

The average excitability was compared between patched D1 and D2-SPNs. A two-way repeated measures ANOVA was used to examine the effect of SPN subtype and depolarization current step on SPN firing frequencies. There was a statistically significant interaction between the SPN subtype and depolarization current (*F*_(4,92)_ = 10.59, *p* < 0.0001). In addition, main effect analysis showed statistical significant effects of SPN subtype (*F*_(1,23)_ = 11.96, *p* = 0.0021) and depolarization current (*F*_(1.7,39.09)_ = 52.26, *p* < 0.0001, ɛ = 0.4249) on SPN AP firing frequency. Multiple comparison analysis with Holm–Šídák's correction showed that the AP firing frequency is significantly higher in D2-SPNs at currents of 200 pA (D2-SPN, *N* = 15, mean ± SEM = 2.8 ± 1.3 Hz vs D1-SPN, *N* = 10, mean ± SEM = 0 ± 0 Hz, adjusted *p* = 0.046), 240 pA (D2-SPN, 6.4 ± 2.3 Hz vs D1-SPN, 0 ± 0 Hz, adjusted *p* = 0.031), 280 pA (D2-SPN, 12.5 ± 2.8 Hz vs D1-SPN, 1.2 ± 0.8 Hz, adjusted *p* = 0.004), 320 pA (D2-SPN, 18.5 ± 2.7 Hz vs D1-SPN, 4.2 ± 2 Hz, adjusted *p* = 0.001), and 360 pA (D2-SPN, 23.6 ± 2.8 Hz vs D1-SPN, 8.2 ± 2.6 Hz, adjusted *p* = 0.002).

The spike threshold was compared between D1 and D2-SPNs using an unpaired *t* test with Welch's correction. One D1-SPN and one D2-SPN had to be excluded because of missing I-V values. D1-SPNs showed a significantly higher spike threshold than D2-SPNs (D1-SPN, *N* = 9, mean ± SD = −56.03 ± 3.82 mV vs D2-SPN, *N* = 14, mean ± SD = −59.96 ± 4.81 mV, *p* = 0.042).

The average input resistance of patched SPNs was compared between D1 and D2-SPN subtypes (Fig. 1*E*). A two-way repeated measures ANOVA was used to compare subtypes at the beginning (first minute) and end of recording (last minute). Both time (*F*_(1,22)_ = 17.27, *p* = 0.0004) and SPN subtype (*F*_(1,22)_ = 6.506, *p* = 0.0182) had a significant effect on the input resistance but there was no interaction effect (*F*_(1,22)_ = 1.486, *p* = 0.2357). Multiple comparison analysis with Bonferroni–Dunn correction showed that the input resistance in D2-SPNs decayed over time (beginning, mean = 97.74 ± 18.5 MΩ vs end, mean = 90.21 ± 16.7 MΩ, *N* = 15; adjusted *p* = 0.0008) but not in D1-SPNs (beginning, mean = 79.18 ± 14.12 MΩ vs end, mean = 75.07 ± 11.13 MΩ, *N* = 9; adjusted *p* = 0.3068). In addition, the input resistance was higher in D2-SPNs at the beginning (97.74 vs 79.19 MΩ, adjusted *p* = 0.0348) but not at the end (90.21 vs 75.07 MΩ, adjusted *p* = 0.12). Although the slight reduction of input resistance was significant, the effect size was small (ω^2^ = 0.023 for within subject comparison calculated as suggested by Lakens, 2013). Neuronal properties were most similar to physiological conditions at the beginning of patch clamp recordings. Overall, the physiological properties of SPNs in these animals were similar to those reported previously (Gertler et al., 2008).

After establishing that patched ChR2-negative neurons were SPNs, IPSCs recorded in response to optogenetic stimulation were assumed to originate from collateral axons of ChR2-positive SPNs. During a 5-s sweep, the 2-ms light pulse triggered a sharp IPSC with short latency (Fig. 1*H*, middle graph). The GABAergic origin of the optically evoked IPSCs was confirmed by blocking the responses with the GABA_A_ antagonist bicuculline (Fig. 1*H*, lower graph). IPSCs were eliminated, confirming that the IPSCs were GABA_A_-mediated synaptic currents. The IPSCs had a prolonged decay time course (Fig. 1*H*, middle graph) caused by the high Cl^–^ internal solution we used to amplify GABA_A_-mediated inhibitory currents. Previous work has shown that high internal Cl^–^ concentrations modulate GABA_A_ channels (Houston et al., 2009) and increase the decay time of IPSCs. In addition, the measured IPSCs are compound IPSCs from multiple presynaptic SPNs. Unitary IPSCs with longer latency hiding in the repolarization tail further increase the IPSC duration.

In summary, patched postsynaptic neurons were identified as SPNs by electrophysiological characteristics. The SPN subtypes showed differences, especially for the shape of the I-V curve and the firing rate in response to current steps. D2-SPNs had a higher input resistance, higher AP frequency and required less injected current to reach threshold than D1-SPNs. APs from ChR2-positive SPNs caused IPSCs in neighboring SPNs with varying amplitude. The IPSCs were GABA_A_ dependent. The high KCl internal solution provided sufficient signal-to-noise ratio for IPSC measurements. These results matched previous reports about differences between SPNs.

### ChR2 expression is specific to SPNs

**Figure 2.**
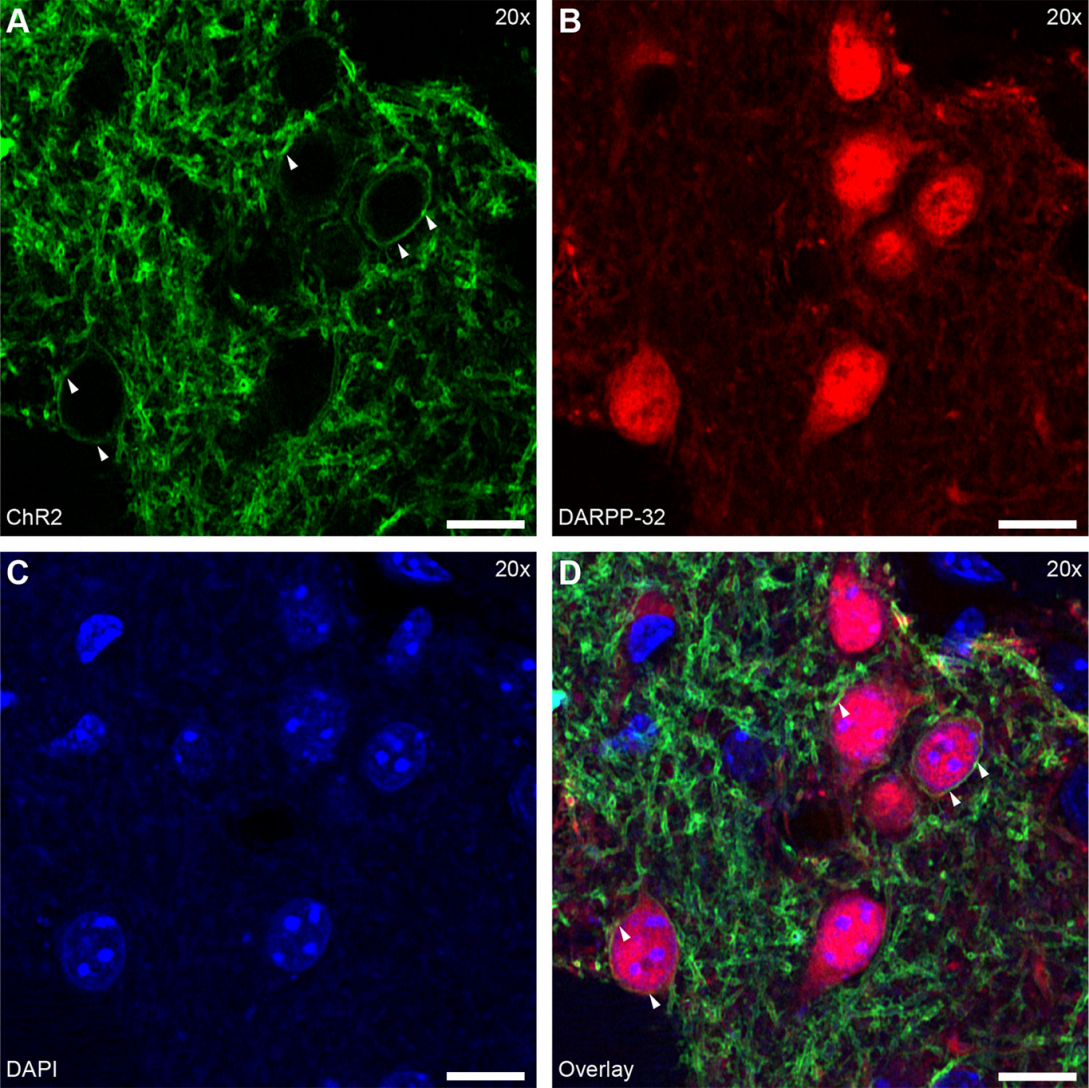
AAV5-mediated ChR2 is expressed in SPNs of D1/A2a-cre mice. ***A–D***, Images of striatal neuropil to show localization of ChR2. ***A***, Cre-dependent expression of ChR2 in cell membranes (arrowheads) in the striatum of A2a-cre mice. ***B***, Spiny neurons labeled with DARPP-32. ***C***, Cell nuclei stained with DAPI. ***D***, Overlay showing DARP-32-positive neurons were also positive for ChR2 (arrowheads). Scale bar: 10 µm.

The specific expression of ChR2 in SPNs was confirmed with immunofluorescence in ChR2-eYFP infected brain slices (Fig. 2). ChR2 was expressed over the entire cell membrane of Cre-positive neurons (Fig. 2*A*). Counterstaining with anti-DARPP-32 for SPNs (Fig. 2*B*) and DAPI for cell nuclei (Fig. 2*C*) revealed colocalization of the SPN marker with the ChR2 signal (Fig. 2*D*, arrowheads). Thus, ChR2 was successfully expressed in SPNs.

**Figure 3.**
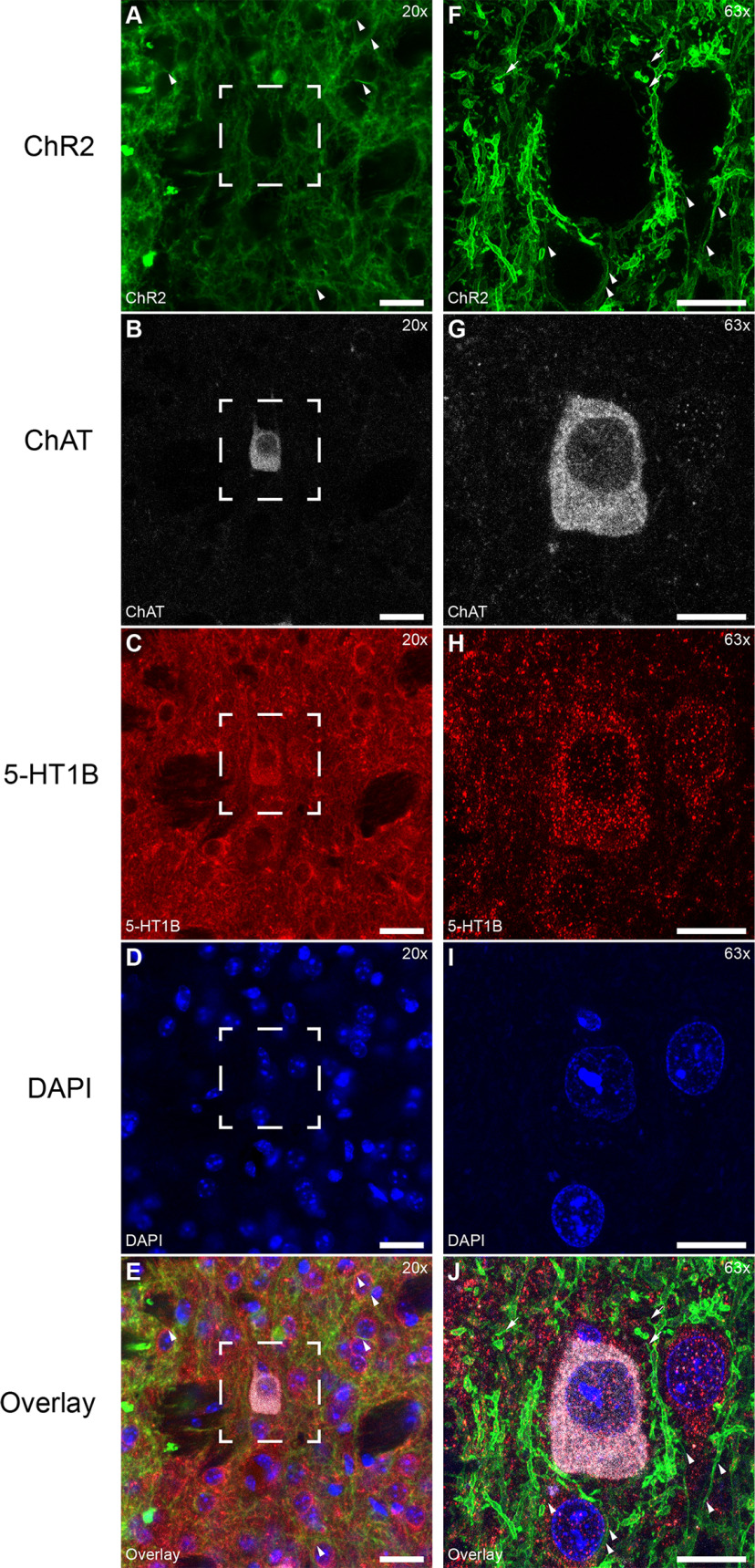
AAV5-mediated ChR2 is absent in CINs of D1/A2a-cre mice. ***A–E***, Different section showing absence of Cre-dependent ChR2 expression from CINs. ***A***, ChR2 expression of D1-SPNs in D1-Cre mice (arrowheads). ***B***, Example CIN. ***C***, Serotonin 5-HT1B receptor clustered on somata of CIN and other neurons. ***D***, Cell nuclei stained with DAPI. Scale bar: 20 µm. ***E***, Overlay of ***E–H***. ***F–J***, Magnification of rectangle from ***A–E***. ***F***, ChR2 expressed in the cell membrane (arrowheads) and spines (arrows) of D1-Cre SPNs. ***G***, CINs. ***H***, Serotonin 5-HT1B receptor. ***I***, Cell nuclei stained with DAPI. ***J***, Overlay showing ChAT-positive cells negative for ChR2 (arrowheads). Serotonin receptor 5-HT1B is clustered on somata of CINs and SPNs. Scale bar: 10 µm.

Earlier studies reported low levels of D1 and D1-like D5 receptors in cholinergic interneurons (CINs; Le Moine et al., 1991; Bergson et al., 1995; Kreitzer, 2009). This could lead in theory to ChR2-positive CINs, which would confound drug effect studies. However, previous studies using Cre-dependent lesion showed no Cre-dependent reduction in CINs or other interneurons in D1 or A2a-Cre mice (Durieux et al., 2009, 2012). Similarly, we did not find Cre-dependent expression of ChR2 in immunolabelled ChAT-positive neurons in D1-Cre mice at an *ex vivo* electrophysiological level with AAV5-DIO-ChR2-eYFP. CINs also express D2 receptors (Alcantara et al., 2003; Wang et al., 2006). To ensure that CINs were not activated optogenetically, we used A2a-Cre mice to target D2-SPNs. ChR2 was expressed in the spines (arrows) and cell membrane (arrowheads) of SPNs (Fig. 3*A*,*F*). Labeling of CINs was revealed with Anti-ChAT (example cell; Fig. 3*B*,*G*). Expression of 5-HT1B (Fig. 3*C*,*H*) produced punctate labeling throughout the neuropil. Cell nuclei are shown by DAPI labeling (Fig. 3*D*,*I*). No expression of ChR2 was observed on CIN cell membranes (Fig. 3*E*,*J*). There was no obvious difference between CINs and SPNs in the concentration of 5-HT1B-positive puncta on the soma. A larger total number of puncta on individual CINs might reflect more synaptic contacts consistent with a larger soma size.

In summary, ChR2 expressing neurons in D1/A2a-Cre mice were SPNs. We confirm that CINs in D1-Cre mice did not express ChR2 at detectable levels and were negative for Cre. This evidence suggests that optically evoked APs were of SPN origin.

### Presynaptic AP firing is reliable and not modified by 5-HT1B receptors

**Figure 4.**
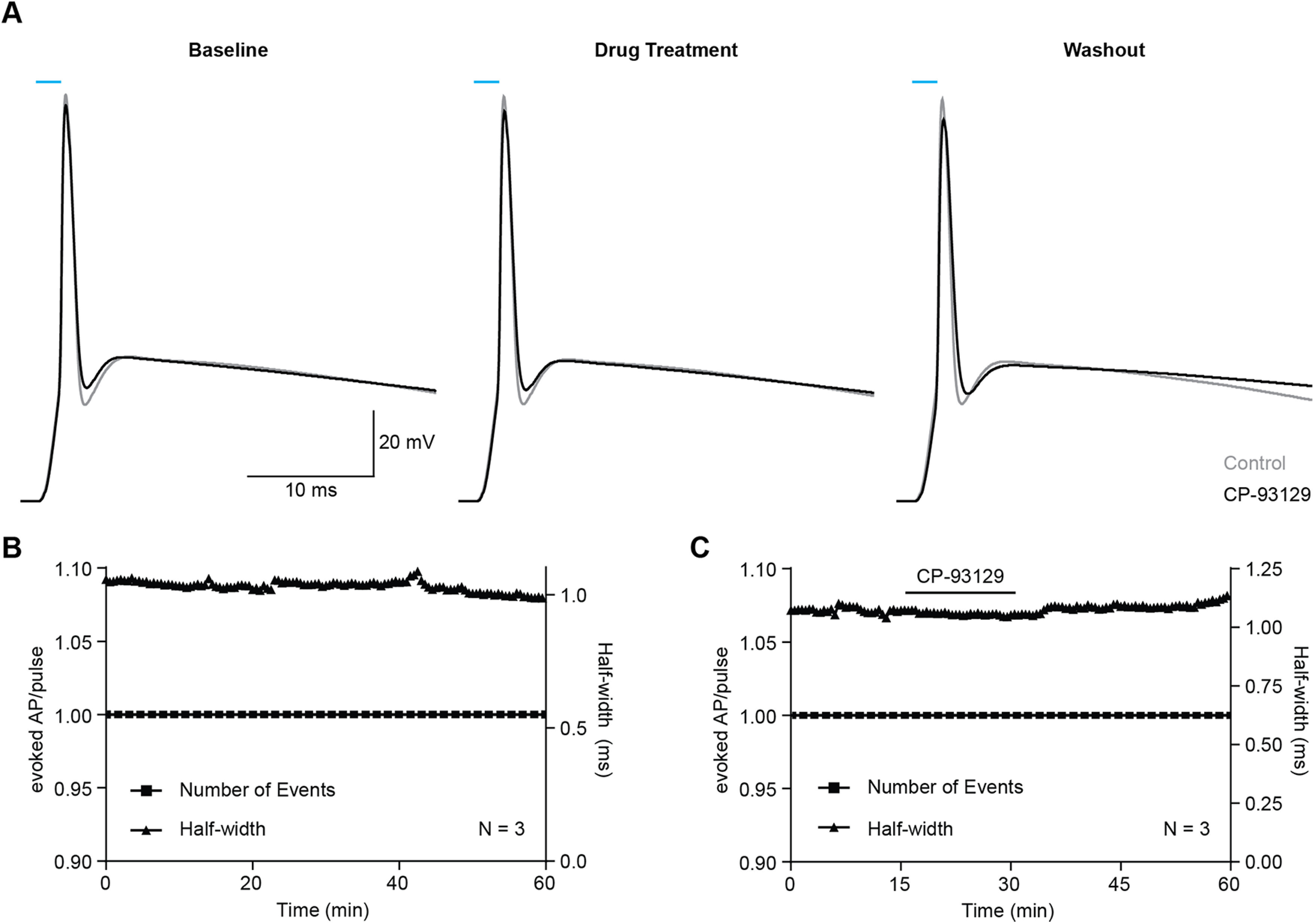
Drug application does not affect ChR2-evoked APs. ***A***, Example AP triggered in a D2-SPNs (D1-Cre mouse) by a 2-ms light pulse before (left), during (middle), and after drug treatment (black traces). Each trace is an average of 180 individual traces. Control APs (gray traces) did not receive CP-93129. ***B***, Effect of repeated AP triggering over time. Triangles, half width; square, occurrence of AP. *N* = 3: D1-Cre (2), A2a-Cre (1). Values are MEAN ± SEM. ***C***, Effect of bath-applied CP-93129. *N* = 3: D1-Cre (3).

To rule out theoretically possible effects of the 5-HT1B receptor on the reliability of presynaptic AP firing, we measured the effect of CP-93129 on the firing behavior of ChR2-positive neurons (Fig. 4). A 2-ms light pulse evoked single APs in control conditions (Fig. 4*A*, gray trace) and had similar effects in the presence of CP-93129 (Fig. 4*A*, black trace), with a constant single AP evoked per light pulse (Fig. 4*B*, lower trace), and stable AP half width (Fig. 4*B*, upper trace). The long, slowly decaying depolarization that follows the AP may be because of the channelrhodopsin we used, ChR2/H134R. This channelrhodopsin has a closing rate of 18–20 ms (Nagel et al., 2005; Lin, 2011). After the LED has turned off this slow closing may have caused a residual postspike depolarization current, thus slowing the return to rest potential (Grossman et al., 2011). Bath application of CP-93129 had no effect on the reliability of AP firing which remained a constant single AP per light pulse over the whole recording time (Fig. 4*C*, lower trace), and did not change the AP half width (Fig. 4*C*, upper trace). These results suggest that CP-93129 had no effect on presynaptic APs or firing reliability of ChR2-positive SPNs. Thus, any drug effects on IPSCs were most likely because of effects on axon terminal and not because of physiological effects at the soma of the presynaptic neuron.

### Serotonin receptor 5-HT1B stimulation blocks lateral inhibition

**Figure 5.**
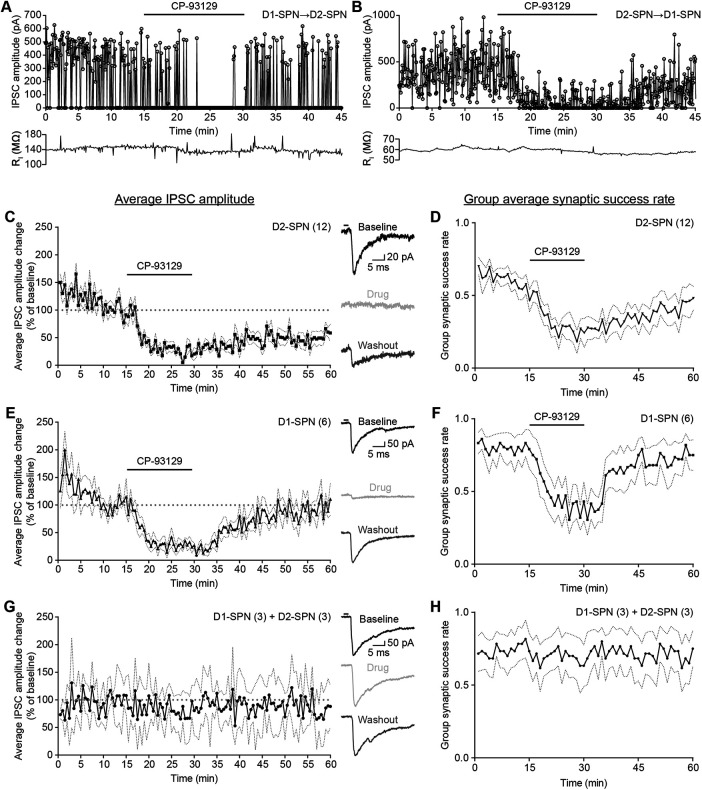
Serotonin receptor 5-HT1B agonist CP-93129 lowers the average IPSC amplitude and synaptic success rate. ***A***, Example IPSC amplitude recording and drug treatment for D1→D2-SPN connections (D1-Cre mouse). Bath application of serotonin receptor 5-HT1B agonist CP-93129 results in silencing of IPSCs. Recording of IPSCs resumes after washout. ***B***, Example IPSC amplitude recording and drug treatment for D2→D1-SPN connections (A2a-Cre mouse). Bath application of serotonin receptor 5-HT1B agonist CP-93129 results in reduction of IPSCs amplitude and increased failures. IPSC recording recovers after washout. ***C***, ***E***, ***G***, Average IPSC amplitude change during drug treatment for D2 SPNs (*N* = 12, D1-Cre mice; ***C***), D1-SPNs (*N* = 6, A2a-Cre mice; ***E***) and control conditions [*N* = 6: D1-Cre (3), A2a-Cre (3); ***G***)]. Each point is an average of six IPSC events. Dotted lines represent SEM IPSC amplitude change was normalized against last 5 min of baseline. The traces on the right are examples recorded during baseline (black), drug treatment/equal period for control conditions (light gray) and washout (dark gray). The black bar represents the 2-ms light pulse. ***D***, ***F***, ***H***, Average group synaptic success rate of D1→D2-SPNs (***D***), D2→D1-SPNs (***F***), and control conditions (***H***). Individual synaptic success rate was calculated as number of recorded IPSCs per minute divided by the number of maximal IPSCs per minute. ***D***, ***F***, ***H***, same cells as ***C***, ***E***, ***G***. Dotted lines represent SEM. All recordings were done in the presence of KA.

To measure the effects of 5-HT1B receptor activation on lateral inhibition, IPSCs were recorded before, during and after application of the serotonin receptor agonist CP-93129 (Fig. 5). Example cells show the effects of bath application of CP-93129 in D1→D2 (Fig. 5*A*) and D2–D1 connections (Fig. 5*B*).

The group averages of the IPSC amplitude (normalized against the mean amplitude over minutes 10–15 as the baseline) showed consistent reduction during drug treatment in D2-SPNs (*N* = 12; Fig. 5*C*). At the same time, the average group synaptic success rate, as a ratio of the number of recorded IPSCs over number of presynaptic stimuli without the amplitude, decreased too (Fig. 5*D*). This suggested a reduction of IPSC amplitude via a shift in synaptic release probability controlled by 5-HT1B receptors. After drug washout, both IPSC amplitude and synaptic success rate made slow recovery without reaching pretreatment levels. The baseline failure rate is rather high compared with that measured using paired recordings, which usually indicates fewer synaptic connections. However, the IPSCs are relatively large, suggesting multiple synaptic connections. When optogenetic excitation is used, it is possible that failures of axonal propagation occur by a different mechanism and frequency than with paired recordings. For example, prolonged subthreshold ChR2 currents causing subthreshold depolarization of an axon might interfere with spike propagation by reducing the availability of sodium currents through inactivation. There is little data on failure rates in SPN connections tested using optogenetics with which to compare. However, even in paired whole cell recordings from SPNs, a wide range of failure rates has been reported. While initial studies described an average success rate of ∼75% (Koos et al., 2004; Taverna et al., 2004), later work showed much lower values of on average 35% and suggested a more complex distribution between different SPN subtypes (Taverna et al., 2008; Planert et al., 2010).

Group averages of normalized IPSC amplitude also showed reduced lateral inhibition after activation of 5-HT1B receptors in D1-SPNs (Fig. 5*E*,*F*). The initial group synaptic success rate was slightly higher than in D2-SPNs. This could indicate more connections between D2→D1-SPNs, if individual synaptic release probability is the same for each SPN subtype. D1-SPNs recovered faster than D2-SPNs and reached baseline level IPSC amplitudes and synaptic success rates. The changes in IPSC amplitude were not because of run-down over time, because responses in untreated slices over 60 min did not show a time-dependent decrease in amplitude (Fig. 5*G*) or group synaptic success rate (Fig. 5*H*). This confirmed that the effect seen in both SPN subtypes is most likely because of effects of CP-93129 on presynaptic 5-HT1B receptors. In summary, CP-93129 acting via 5-HT1B receptors reduced lateral inhibition between SPNs, in part if not wholly by increasing the synaptic failure rate.

**Figure 6.**
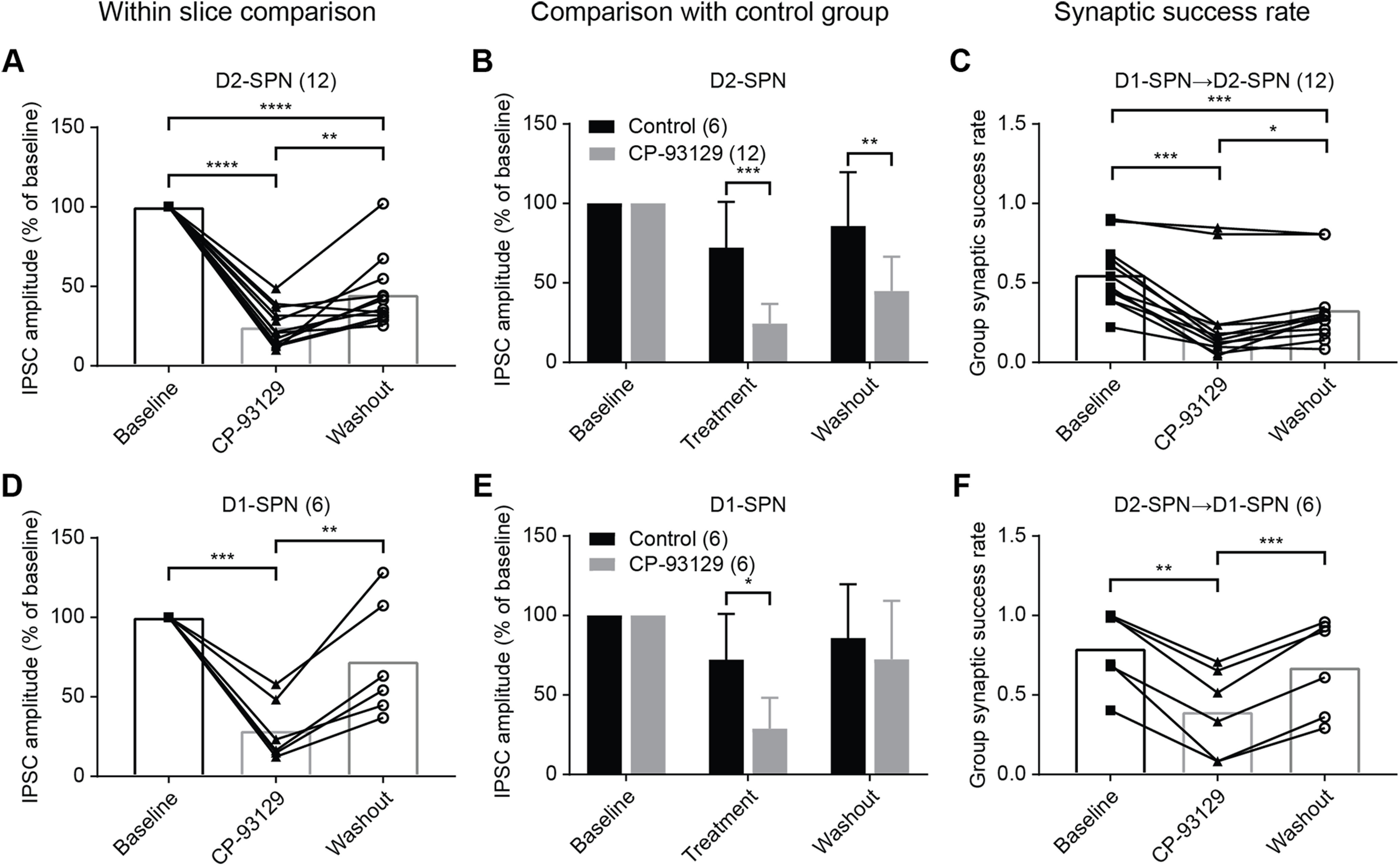
CP-93129 changes average IPSC amplitude and synaptic release probability in local axon collaterals. ***A***, Statistical analysis of average IPSC amplitude change in D2-SPNs (D1-Cre mice) during baseline, drug treatment, and washout (*N* = 12). ***B***, Comparison of average IPSC amplitude of drug treatment in D2-SPNs (D1-Cre mice, *N* = 12) with control group (*N* = 6). Mean ± SD. ***C***, Analysis of average group synaptic success rate for D1→D2-SPN connections (D1-Cre mice, *N* = 12). ***D***, Statistical analysis of average IPSC amplitude change in D1-SPNs (A2a-Cre mice) during baseline, drug treatment, and washout (*N* = 6). ***E***, Comparison of average IPSC amplitude of drug treatment in D1-SPNs (A2a-Cre mice, *N* = 6) with control group (*N* = 6). Mean ± SD. ***F***, Analysis of average group synaptic success rate for D2→D1-SPN connections (A2a-Cre mice, *N* = 6). ns, *p* > 0.05; *p < 0.05, **p < 0.01, ***p < 0.001, ****p < 0.0001.

Statistical analyses of the effects of CP-93129 are shown in Figure 6. First, the group average of the IPSC amplitude was compared during pretreatment, CP-93129 and washout. The pretreatment phase was the first 15 min of recording before drug application and considered as baseline. Five minutes from baseline (10–15), CP-93129 (25–30) and washout (40–45) were averaged for comparison. The selected period of 5 min represented the most stable time of each recording phase, taking into account the time for the neuron adjust to the internal solution, drug diffusion in the bath, and drug washout. A one-way ANOVA for repeated measures showed a significant effect of CP-93129 on the IPSC amplitude, compared with baseline, for D2-SPNs (F_(1.523, 16.75)_ = 110.5, *p* < 0.0001; Holm–Šídák's correction for multiple comparison: baseline–CP-93129 *p* < 0.0001, baseline–washout *p* < 0.0001; CP-93129–washout *p* = 0.0038; Fig. 6*A*) and D1-SPNs (F_(1.140, 5.699)_ = 21.28, *p* = 0.0037; Holm–Šídák's correction for multiple comparison: baseline–CP-93129 *p* = 0.0008, baseline–washout *p* = 0.1262; CP-93129–washout *p* = 0.0079; Fig. 6*D*). Unlike the washout, the reduction of IPSC amplitude was similar for both D1→D2 and D2→D1 connections. D1→D2 connections recovered partially during 15 min of washout. The IPSC amplitude increase was significantly different from the drug phase but lower than baseline. Conversely, D1-SPNs recovered to baseline levels on average (and in one case above) after the washout. Comparison of washout in D1→D2 and D2→D1 connections (Fig. 5*C*,*E*) showed that the recovery of functional lateral connectivity takes longer in D2-SPNs than D1-SPNs. The reason for differences in the recovery is unclear but may be a difference between the different transgenic strains used in the two conditions.

Second, a comparison of IPSC amplitude with the control group was made. The data were analyzed in the same way as the within slice comparison. Matching periods were chosen from the control group for averaging (10–15, 25–30, 40–45 min) and normalized against baseline. Some differences were seen between the corresponding treatment and washout phases and baseline (Fig. 6*B*,*E*) because of normalization. There was no significant difference between phases in the control group. D2-SPNs showed a smaller average IPSC amplitude during CP-93129 application and washout compared with the control group (multiple *t* tests corrected with Holm–Šídák's method, treatment–control adjusted *p* = 0.0002, washout–control *p* = 0.0013; Fig. 6*B*). In contrast, the IPSC amplitude in D1-SPNs was reduced by CP-93129 but recovered completely during washout (multiple *t* tests, treatment–control *p* = 0.0029, washout–control *p* = 0.3418; Fig. 6*E*). The results were similar to the within slice comparison. The difference in amplitude during washout confirmed the observation that the recovery of lateral inhibition is less in D1→D2 than in D2→D1 connections.

The average synaptic success rate was analyzed in the same way. Averages were compared from baseline, CP-93129 and washout (minutes 10–15, 25–30, and 40–45). There was a significant reduction in synaptic success rate after drug application for D2-SPNs (Fig. 6*C*). The success rate recovered after washout but was lower than baseline levels (repeated measures one-way ANOVA F_(1.389, 15.28)_ = 38.24 *p* < 0.0001; Tukey correction for multiple comparison: baseline–CP-93129 *p* = 0.0001; baseline–washout *p* = 0.0001; CP-93129–washout *p* = 0.0213). In contrast, D1-SPNs showed a significant reduction in average synaptic success rate, which recovered completely (repeated measures one-way ANOVA F_(1.520, 7.598)_ = 46.71, *p* < 0.0001; Tukey correction for multiple comparison: baseline–CP-93129 *p* = 0.0013; baseline–washout *p* = 0.0941; CP-93129–washout *p* = 0.0005; Fig. 6*F*). These results matched the effect on the IPSC amplitude and confirmed that 5-HT1B activation through CP-93129 acts at least in part by reducing the synaptic release probability of GABA. These data, however, cannot exclude the possibility of an effect on quantal size, because the optically evoked IPSCs are compounds composed of an unknown number of synaptic events that vary depending on the number of synaptic connections activated and the failure rate. Thus, a change in quantal size leading to a reduction in optically-evoked IPSC amplitude would be indistinguishable from a reduction in optically-evoked IPSC amplitude caused by a lower release probability and fewer unitary synaptic events.

In summary, the statistical analysis confirmed the effect of 5-HT1B activation by CP-93129 on IPSC amplitude in both D1→D2 and D2→D1 connections. This effect was mediated at least partially by a change in release probability on the presynaptic side, which reduced the synaptic success rate and IPSC amplitude.

### Effects of 5-HT1B activation on long-latency or spontaneous IPSCs

**Figure 7.**
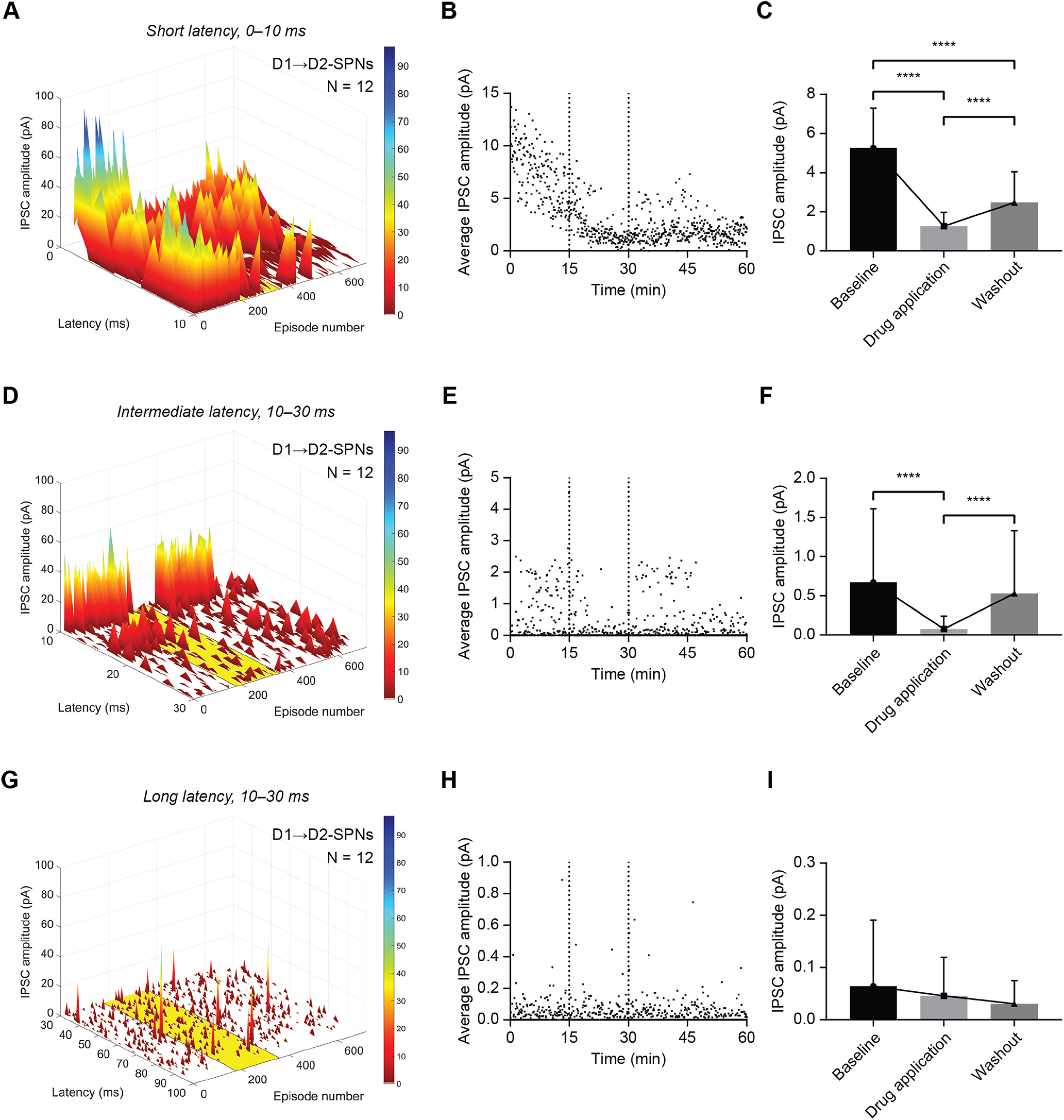
CP-93129 affects ChR2-evoked IPSCs in D2-SPNs up to 30 ms after optical stimulation. ***A***, Average distribution and amplitude of IPSCs recorded in D2-SPNs (D1-Cre mice) within the first 10 ms after the light stimulus. The yellow area indicates application of CP-93129. Each episode equals 5 s. ***B***, Average IPSC amplitude of all recorded IPSCs in D2-SPNs (D1-Cre mice) with a latency of ≤10 ms over 60 min. The two dotted lines mark the application of CP-93129. ***C***, Statistical analysis of average IPSC amplitude within 10 ms after the light pulse in D2-SPNs (D1-Cre mice) during baseline, drug treatment, and washout. Values are mean ± SD. ***D***, Average distribution and amplitude of IPSCs recorded in D2-SPNs (D1-Cre mice) between 10 and 30 ms after the light stimulus. The yellow area indicates application of CP-93129. Each episode equals 5 s. ***E***, Average IPSC amplitude of all recorded IPSCs in D2-SPNs (D1-Cre mice) with a latency between 10 and 30 ms over 60 min. The two dotted lines mark the application of CP-93129. ***F***, Statistical analysis of average IPSC amplitude between 10 and 30 ms after the light pulse in D2-SPNs (D1-Cre mice) during baseline, drug treatment, and washout. Values are mean ± SD. ***G***, Average distribution and amplitude of IPSCs recorded in D2-SPNs (D1-Cre mice) between 30 and 100 ms after the light stimulus. The yellow area indicates application of CP-93129. Each episode equals 5 s. ***H***, Average IPSC amplitude of all recorded IPSCs in D2-SPNs (D1-Cre mice) with a latency between 30 and 100 ms over 60 min. ***I***, Statistical analysis of average IPSC amplitude between 30 and 100 ms after the light pulse in D2-SPNs (D1-Cre mice) during baseline, drug treatment, and washout. Values are mean ± SD; *N* = 12. ns, *p* > 0.05; *p < 0.05, **p < 0.01, ***p < 0.001, ****p < 0.0001.

In the foregoing analysis, the effect of CP-93129 on IPSCs was tested using measures of IPSCs that occurred within 10 ms of the optical stimulus. The IPSCs that occur outside that time interval could be caused by later presynaptic spikes in the axons of presynaptic SPNs, spontaneous release from GABA interneurons and spontaneous or miniature IPSCs from other SPNs. The effects of 5-HT1B receptor activation on longer latency or spontaneous IPSCs was addressed by analyzing all IPSCs over a period of 100 ms after the light pulse, in three timeframes: 0–10, 10–30, and 30–100 ms (Fig. 7). This yielded a group average IPSC amplitude at a given time after the stimulation. The procedure highlights IPSCs that repeatedly occur at the same latency. Time-locked events thus have a higher average amplitude while spontaneous, random events are less likely to appear at the exact same time resulting in smaller amplitudes. In addition, the average of all IPSCs within 10 ms after the light pulse in this episode was plotted in a separate graph. This average can represent either large single IPSC or multiple smaller IPSCs for example from more than one presynaptic event.

The effect of CP-93129 on IPSCs in D2 neurons (*N* = 12) that occurred within 10 ms of the light pulse are shown in Figure 7*A–C*. Most events in this 10-ms period after optical stimulation were expected to be synaptic responses to presynaptic APs in D1 neurons. The IPSCs in D2 neurons occurred with variable latency (Fig. 7*A*). After a 15-min baseline, application of CP-93129 caused a decrease in the average IPSC amplitude and an increase in the number of failures (Fig. 7*A*,*B*). For statistical analysis, data points from 5 min of baseline (10–15), CP-93129 (25–30), and washout (40–45) were averaged as described before. Repeated measures one-way ANOVA showed significant differences of average IPSC amplitude for D2-SPNs, confirming the effect of CP-93129 for IPSCs within 10 ms after the light pulse (F_(1.609, 94.94)_ = 104.4, *p* < 0.0001; Holm–Šídák's correction for multiple comparison: baseline–CP-93129 *p* < 0.0001, baseline–washout *p* < 0.0001; CP-93129–washout *p* < 0.0001; Fig. 7*C*). During washout, the average IPSC amplitude did not recover to baseline levels (see episode 400–720), and some eliminated events did not return at the same latency.

Second, IPSCs with latencies of 10–30 ms were analyzed. Application of CP-93129 caused a reduction of average IPSC amplitude particularly in those IPSCs with around 12-ms latency (Fig. 7*D*). The average distribution of IPSCs for D2-SPNs was reduced by CP-93129 (yellow rectangle, episode 180–360) and recovered afterward (Fig. 7*D*,*E*). Repeated measures one-way ANOVA for D2-SPNs (Fig. 7*F*) showed significant differences in average IPSC amplitude between baseline, CP-93129, and washout (F_(1.465, 86.46)_ = 10.80, *p* = 0.0003; Holm–Šídák's correction for multiple comparison: baseline–CP-93129 *p* < 0.0001; baseline–washout *p* = 0.3957; CP-93129–washout *p* < 0.0001).

Third, IPSCs in the period 30–100 ms after optical stimulation were analyzed. Events measured in this period are not related to optogenetic stimulation and were most likely because of spontaneous activity from interneurons or spontaneous release events from SPNs or interneurons. The IPSCs occurring in this period in D2-SPNs (Fig. 7*G*) were not affected by CP-93129. The amplitude average over latency for each episode showed no effect of CP93129 (between dotted lines) for D2-SPNs (Fig. 7*H*). Repeated measures one-way ANOVA of the average IPSC amplitude confirmed no difference between baseline, CP-93129, and washout for D2-SPNs (F_(1.492, 88.01)_ = 2.087, *p* = 0.1424; Holm–Šídák's correction for multiple comparison: baseline–CP-93129 *p* = 0.6006; baseline–washout *p* = 0.1537; CP-93129–washout *p* = 0.3643; Fig. 7*I*). The lack of effect of CP-93129 on the IPSCs occurring 30–100 ms after optical stimulation may indicate that they originate from a different subpopulation than SPNs, and most probably represented IPSCs from spontaneously active interneurons or spontaneous release events from those neurons such as miniature IPSCs.

**Figure 8.**
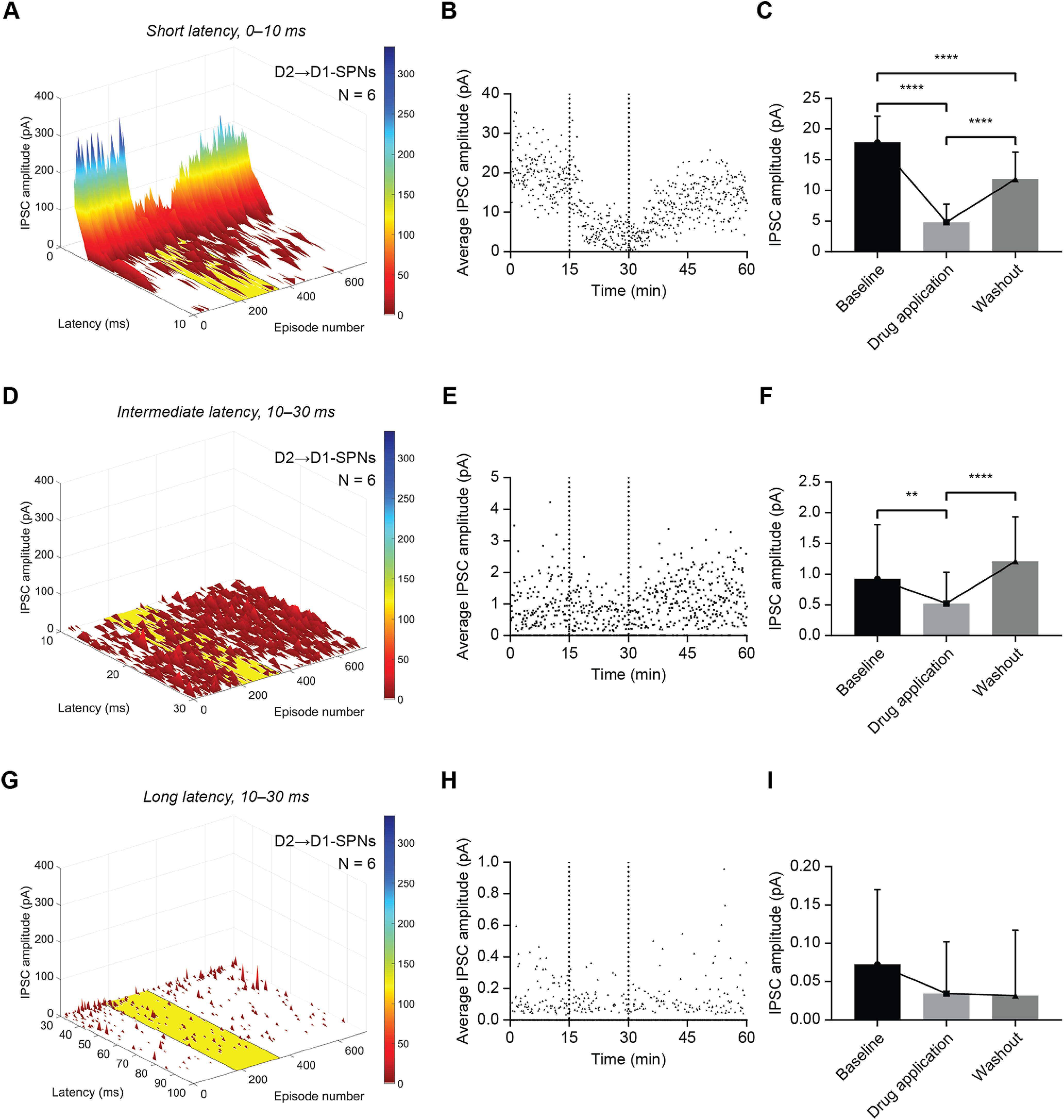
CP-93129 affects ChR2-evoked IPSCs in D1-SPNs up to 30 ms after optical stimulation. ***A***, Average distribution and amplitude of IPSCs recorded in D1-SPNs (A2a-Cre mice) within the first 10 ms after the light stimulus. Note the higher average amplitude compared with D2-SPNs. The yellow area indicates application of CP-93129. Each episode equals 5 s. ***B***, Average IPSC amplitude of all recorded IPSCs in D1-SPNs (A2a-Cre mice) with a latency of ≤10 ms over 60 min. The two dotted lines mark the application of CP-93129. ***C***, Statistical analysis of average IPSC amplitude within 10 ms after the light pulse in D1-SPNs (A2a-Cre mice) during baseline, drug treatment, and washout. IPSC recovery did not reach baseline levels. Values are mean ± SD. ***D***, Average distribution and amplitude of IPSCs recorded in D1-SPNs (A2a-Cre mice) between 10 and 30 ms after the light stimulus. The yellow area indicates application of CP-93129. Each episode equals 5 s. ***E***, Average IPSC amplitude of all recorded IPSCs in D1-SPNs (A2a-Cre mice) with a latency between 10 and 30 ms over 60 min. The two dotted lines mark the application of CP-93129. ***F***, Statistical analysis of average IPSC amplitude between 10 and 30 ms after the light pulse in D1-SPNs (A2a-Cre mice) during baseline, drug treatment, and washout. Values are mean ± SD. ***G***, Average distribution and amplitude of IPSCs recorded in D1-SPNs (A2a-Cre mice) between 30 and 100 ms after the light stimulus. Each episode equals 5 s. ***H***, Average IPSC amplitude of all recorded IPSCs in D1-SPNs (A2a-Cre mice) with a latency between 30 and 100 ms over 60 min. ***I***, Statistical analysis of average IPSC amplitude between 30 and 100 ms after the light pulse in D1-SPNs (A2a-Cre mice) during baseline, drug treatment, and washout. Values are mean ± SD; *N* = 6. ns, *p* > 0.05; *p < 0.05, **p < 0.01, ***p < 0.001, ****p < 0.0001.

The effect of CP-93129 on IPSCs recorded in D1-SPNs (*N* = 6) was similar to the effect on IPSCs recorded in D2-SPNs (Fig. 8). Some differences in the properties of the D2→D1 IPSCs from the D1→D2 IPSCs were evident in the baseline responses in the first 10-ms period (Fig. 8*A*,*B*). The IPSCs recorded in D1-SPNs were precisely time locked to the stimulus, unlike those in D2-SPNs. The IPSCs also had a several-fold higher average amplitude in D1-SPNs than in D2-SPNs. Application of CP-93129 again caused reduction in the mean amplitude of the IPSCs, particularly obvious in the short-latency IPSCs occurring in the first few milliseconds after optical stimulation (Fig. 8*A*). The recovery of the IPSC amplitude during washout of the drug was faster than for D2-SPNs and almost returned to baseline levels. Statistically, CP-93129 significantly reduced IPSCs recorded in D1-SPNs (F_(1.989, 117.4)_ = 150.7, *p* < 0.0001; Holm–Šídák's correction for multiple comparison: baseline–CP-93129 *p* < 0.0001; baseline–washout *p* < 0.0001; CP-93129–washout *p* < 0.0001; Fig. 8*C*).

Analysis of IPSCs in the second time window (10–30 ms) showed that IPSCs in D1-SPNs were reduced slightly by CP-93129 (Fig. 8*D*). The same results were found in the amplitude average over the latency for each episode (Fig. 8*E*). There were some IPSCs within 10–30 ms after the light pulse, which were affected by CP-93129. These IPSCs can originate from secondary presynaptic APs, antidromic spikes or deep SPNs, which receive less light resulting in irregular firing, or innervations to distant dendritic synapses. Statistical analysis for D1-SPNs also result in significant differences (F_(1.887, 111.3)_ = 14.21, *p* < 0.0001; Holm–Šídák's correction for multiple comparison: baseline–CP-93129 *p* = 0.0099; baseline–washout *p* = 0.1163; CP-93129–washout *p* < 0.0001; Fig. 8*F*). Taken together, the results were similar to the effects in the first 10-ms period.

In the 30- to 100-ms time window, CP-93129 had very little effect on IPSCs in D1-SPNs (Fig. 8*G*,*H*). A repeated measures one-way ANOVA suggested a significant overall effect (F_(1.845, 108.9)_ = 3.897, *p* = 0.0262; Fig. 8*I*). This is because of the series of IPSCs close to 30 ms. Subsequent multiple comparison showed no significant differences (Holm–Šídák's correction for multiple comparison: baseline–CP-93129 *p* = 0.0553; baseline–washout *p* = 0.0756; CP-93129–washout *p* = 0.9792). Taken together, IPSCs recorded 30–100 ms after an AP were not affected by CP-93129. This suggested that 5-HT1B-expressing presynaptic terminals of lateral SPNs did not mediate the events in this period. Additionally, the latency of IPSCs still triggered by SPN-SPN lateral inhibition might be higher for D1-SPNs.

In summary, the analysis of the effect of CP-93129 on IPSC with respect to latency showed IPSCs of unspecific origin, which are not affected by 5-HT1B activation and not caused by lateral inhibition between SPNs. The influence of unspecific events did not confound the effect of CP-93129 and was most prominent among IPSCs with latencies of >30 ms. The lateral IPSCs fell mostly within the first 10 ms after the triggered AP with some secondary events with 10–30 ms. There were clear differences between the SPN subtypes. D1→D2 connections on average had a lower IPSC amplitude and time-locked secondary events while D2→D1 SPNs had a much higher average IPSC amplitude and large variability in secondary IPSC latency. However, short latency IPSCs in both D1 and D2-SPNs were significantly reduced by CP-93129.

### IPSCs from fast-spiking interneurons (FSIs) are not affected by 5-HT1B activation

**Figure 9.**
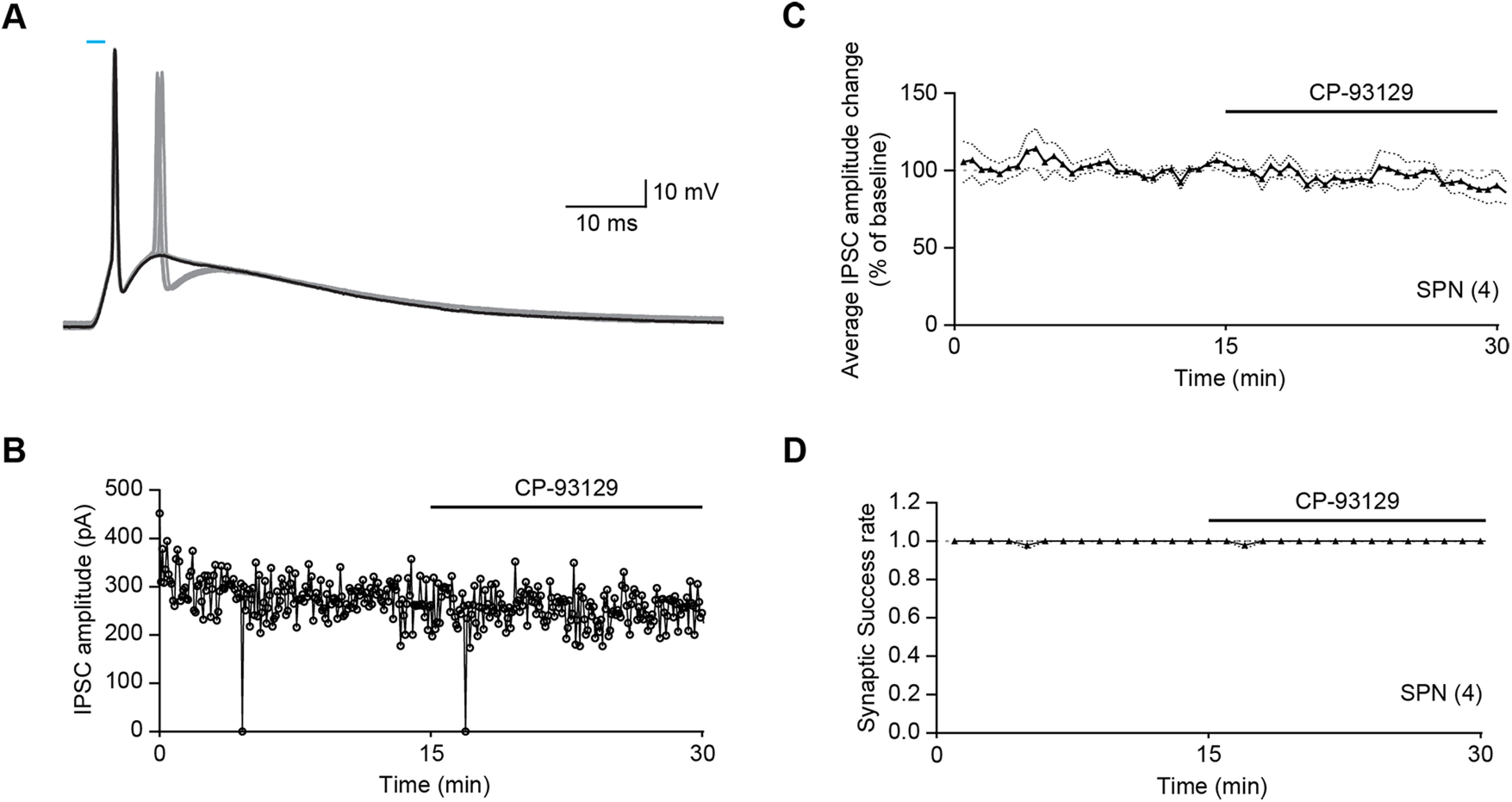
CP-93129 does not affect ChR2-IPSCs from FSIs. ***A***, Example AP triggered in a FSI (PV-Cre mouse) by a 2-ms light pulse. Optical stimulation resulted in one (black) or two APs (gray). Blue light bar represents LED stimulus. ***B***, Example IPSC amplitude recording and drug treatment for FSI→SPN connections (PV-Cre mouse). Bath application of serotonin receptor 5-HT1B agonist CP-93129 has no effect on IPSCs. ***C***, Average IPSC amplitude change during CP-93129 treatment in PV-Cre mice (*N* = 4). Horizontal line at 100% was added as visual guideline. Dotted lines represent SEM IPSC amplitude change was normalized against minutes 10–15 (baseline). Each point is an average of six IPSC events. ***D***, Average group synaptic success rate of SPNs with CP-93129. Dotted lines represent SEM. All recordings were done in the presence of KA.

To further investigate whether unspecific events might in part come from FSIs that are not affected by 5-HT1B, optical stimulation was applied to PV-Cre mice injected with ChR2. A 2-ms light pulse resulted in one to two APs followed by a slowly decaying depolarization tail (Fig. 9*A*). Optically evoked IPSCs from FSI were recorded in a subset of SPNs. Bath application of CP-93129 had no effect on the individual and normalized, average IPSC amplitude (Fig. 9*B*,*C*). The synaptic success rate in FSI→SPN connections was very high, consistent with previous reports (Koos et al., 2004). This high synaptic success rate did not change after adding CP-93129 (Fig. 9*D*).

### Specificity of CP-93129 effects on IPSCs

**Figure 10.**
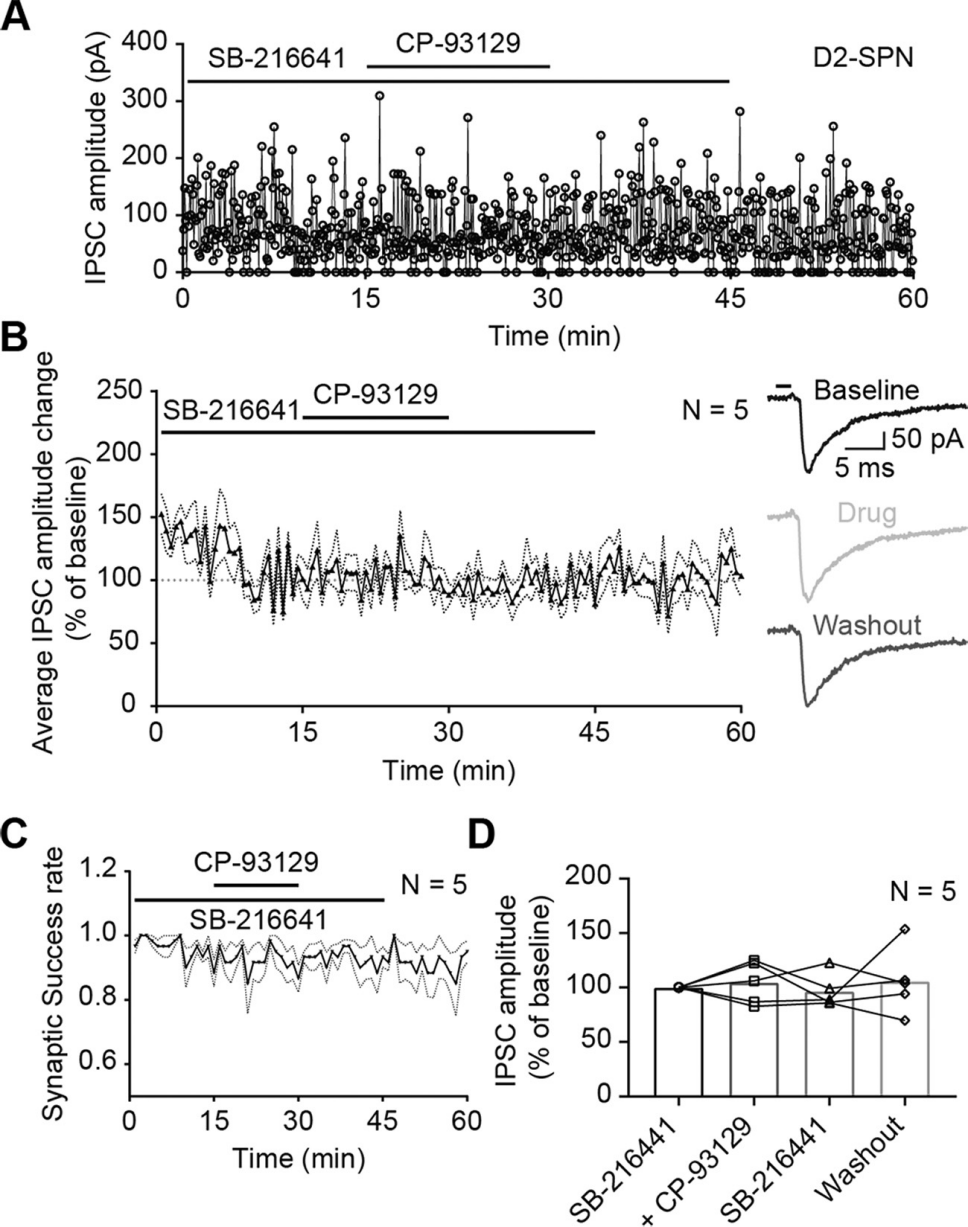
Optical-evoked APs are somatic. ***A***, Example recording of IPSC amplitudes in a D1-SPN (A2a-Cre mouse) in the presence of sodium channel blocker TTX (0.5 µm). ***B***, Average IPSC amplitude change during TTX treatment in D1-Cre and A2a-Cre mice [*N* = 4: D1-Cre (1), A2a-Cre (3)]. Horizontal line at 100% was added as visual guideline. Dotted lines represent SEM IPSC amplitude change was normalized against minutes 10–15 (baseline). Each point is an average of six IPSC events. The traces on the right are examples recorded during baseline (black), drug treatment (light gray), and CP-93129 washout (dark gray). The black bar represents the 2-ms light pulse. ***C***, Average group synaptic success rate of SPNs with TTX. Dotted lines represent SEM. ***D***, Before and after plot of average IPSC amplitude change of SPNs with TTX. Bars represent the mean. All recordings were done in the presence of KA.

To test whether IPSCs were triggered by somatic AP or direct excitation of presynaptic terminals, optical stimulation was applied in the presence of TTX (0.5 µm; Fig. 10*A*). TTX is a well-established sodium channel blocker and prevents AP propagation down the axon. The application of TTX abolished all IPSCs, which started to recover only after a prolonged washout phase. This was confirmed in the group average (*N* = 4; Fig. 10*B*). The average synaptic success rate declined immediately after TTX entered the bath (Fig. 10*C*). The recovery of the synaptic success rate in the second half of the recording session was more prominent than for the IPSC amplitude because both unitary and multiple contemporaneous events after the light pulse are detected as a single IPSC. Repeated measures one-way ANOVA showed significant reduction of IPSC amplitude after TTX application (F_(1.120, 3.360)_ = 481.9, *p* < 0.0001; Holm–Šídák's correction for multiple comparison: baseline–TTX *p* < 0.0001; Fig. 10*D*). This suggested that IPSCs originate from presynaptic APs or ChR2-positive cut axons. While this shows that IPSCs were not caused by direct excitation of ChR2-positive terminals, abolition of the IPSCs by TTX does not rule out a contribution of ChR2 excitation of cut axons to IPSCs. On the other hand, experimental (Hass and Glickfeld, 2016) and theoretical (Foutz et al., 2012) studies indicate that activation of axons with ChR2 has a much higher threshold irradiance than activation of soma-dendritic regions, thus a higher intensity of light is needed to excite cut axons compared with cell bodies. The light level used in the present experiments was highly attenuated and therefore probably not sufficient to trigger spikes in cut axons. In support of this, we did not see any antidromic axon-initiated spikes which might have been the case if axons were excited to suprathreshold levels by the light stimulation. The absence of miniature IPSCs in the presence of TTX was probably because of their rare occurrence in SPNs (Ade et al., 2008) and the short interval analyzed (10 ms).

**Figure 11.**
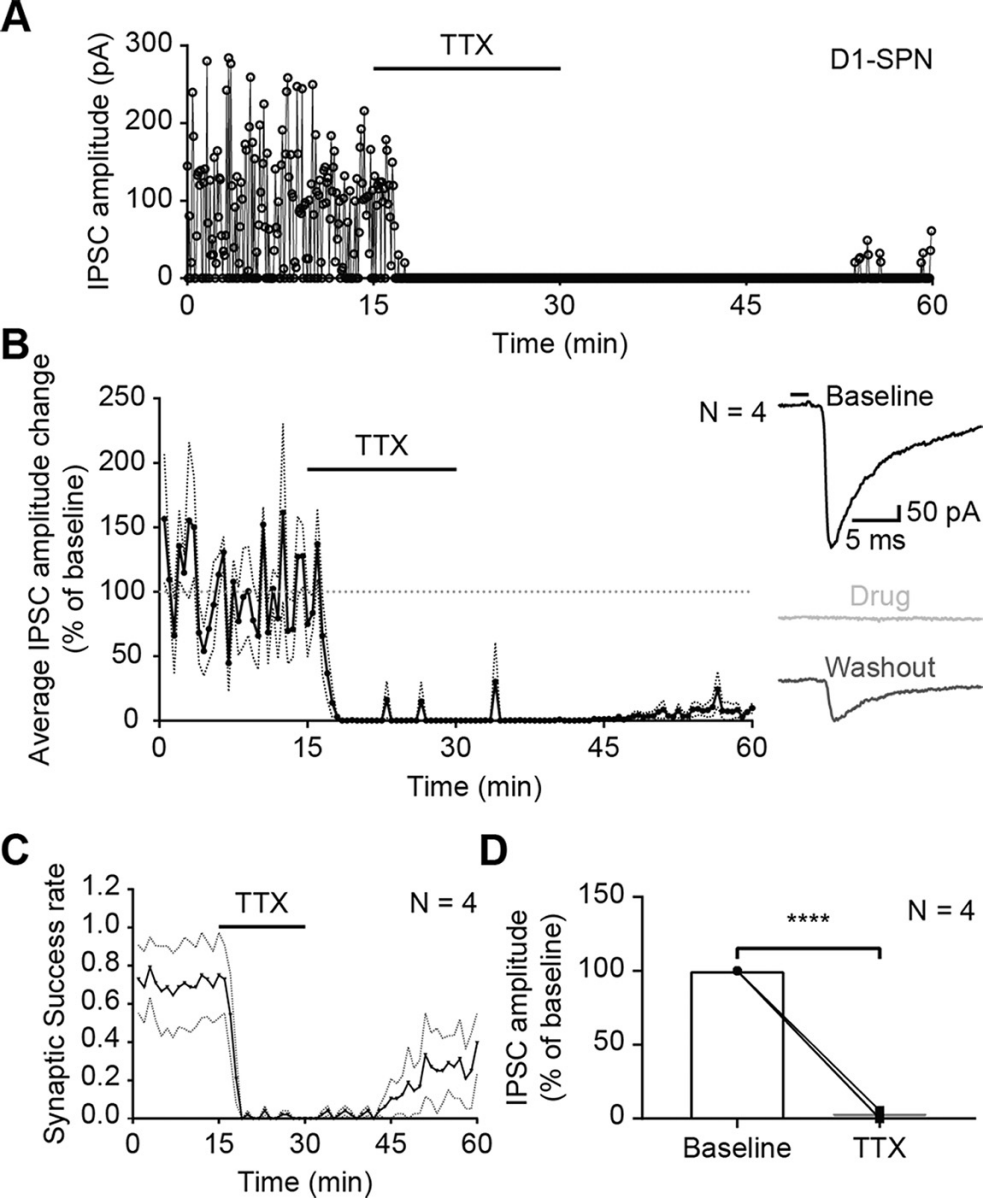
CP-93129 acts via 5-HT1B receptors located on presynaptic SPN terminals. ***A***, Example recording of IPSC amplitudes in a D2-SPN (D1-Cre mouse) with serotonin receptor antagonist SB-216641 and agonist CP-93129. Recording started in the presence of SB-216641 and CP-93129 was added to the bath after 15 min. ***B***, Average IPSC amplitude change in D2-SPNs (D1-Cre mice) during drug treatment (*N* = 5). Horizontal line at 100% was added as visual guideline. Each point is an average of six IPSC events. Dotted lines represent SEM IPSC amplitude change was normalized against minutes 10–15 (baseline). The traces on the right are examples recorded during baseline (black), drug treatment (light gray), and washout (dark gray). The black bar represents the 2-ms light pulse. ***C***, Average group synaptic success rate of D2-SPNs (D1-Cre mice) with SB-216641 and CP-93129. Dotted lines represent SEM. ***D***, Before and after plot of average IPSC amplitude change of D2-SPNs (D1-Cre mice) during drug treatment. Bars represent the mean. All recordings were done in the presence of KA. ns, *p* > 0.05; *p < 0.05, **p < 0.01, ***p < 0.001, ****p < 0.0001.

The agonist CP-93129 is a highly specific and widely used agonist for 5-HT1B receptors. Previous studies showed successful use in slice experiments at concentrations similar to those used here (Mathur et al., 2011; Huang et al., 2013). In order to confirm a specific action at 5-HT1B receptors, and rule out other explanations for the effect on IPSC amplitude, CP-93129 was tested in the presence of serotonin receptor 5-HT1B antagonist SB-216641 (Fig. 11; Price et al., 1997). The antagonist SB-216641 (10 µm) was added to ACSF 5 min before the recording. Bath application of CP-93129 followed after 15 min and was washed out after another 15 min. The antagonist was removed after 45 min of recording (Fig. 11*A*). There was no visible effect of the agonist in the presence of the antagonist. The antagonist had also no immediate effect on the IPSC amplitude either. The normalized group average (*N* = 5) showed the same results: the effect of CP-93129 on the IPSC amplitude was blocked when the agonist and antagonist were applied together (Fig. 11*B*). The washout of the antagonist SB-216641 also had no effect. The synaptic success rate was calculated as described above and did not change (Fig. 11*C*). There was no difference between SB-216641 and SB-216641 plus CP-93129 or pure ACSF. Again, comparing the average of the last 5 min of each recording phase revealed no difference (Fig. 11*D*). Repeated measures one-way ANOVA showed no significant effect of any treatment phase (F_(1.830, 7.319)_ = 0.2285, *p* = 0.7831; Holm–Šídák's correction for multiple comparison: baseline SB-216641–SB-216641 plus CP-93129 *p* = 0.9869, baseline SB-216641–SB-216641 *p* = 0.9869, baseline SB-216641–washout *p* = 0.9869, SB-216641 plus CP-93129–SB-216641 *p* = 0.9765, SB-216641 plus CP-93129–washout *p* = 09,869, SB-216641–washout *p* = 0.9869). This indicated that the effect of CP-93129 on IPSC amplitudes was mediated by 5-HT1B receptors. Furthermore, SB-216641 had no effect on IPSC amplitude.

In summary, these results showed that the recorded IPSCs were triggered by somatic APs from ChR2-positive SPNs and CP-93129 was acting through 5-HT1B receptors to reduce IPSC amplitudes.

## Discussion

The main finding of this study is the reduction of lateral inhibition between striatal SPNs by serotonin. In the past, several groups have shown lateral inhibitory interactions among SPNs with paired recordings (Czubayko and Plenz, 2002; Tunstall et al., 2002; Koos et al., 2004; Venance et al., 2004; Taverna et al., 2008; Planert et al., 2010) and optical stimulation (Chuhma et al., 2011). Other groups have shown that 5-HT1B receptors modulate the release of GABA from the synapses of SPNs in projection areas (Johnson et al., 1992; Stanford and Lacey, 1996; Hoyer et al., 2002; Sari, 2004). Our results are the first to show that serotonin 5-HT1B receptors modulate lateral inhibition in the striatum. Stimulation of 5-HT1B receptors caused a decrease in IPSCs evoked by optical stimulation of SPNs. The effects of 5-HT1B receptors were seen both in synapses connecting D1 to D2-SPNs and in D2 to D1-SPN connections. The effects appeared to involve a decrease in the probability of GABA release by a presynaptic action of the receptors. Previous work showed the involvement of serotonin in the striatum in long-term depression and glutamatergic input from the cortex and thalamus, which acts via presynaptic 5-HT1B receptors and 5-HT4 receptors on SPNs (Mathur et al., 2011; Huang et al., 2013; Cavaccini et al., 2018). The present results extend understanding of the complex effects of serotonin in the striatum, by showing that 5-HT1B receptors are also present on collateral axons of SPNs and play an important role in striatal signaling. In particular, our results suggest a new role for serotonin in the function of the striatum, as a modulator of lateral inhibition

In the present study, optical stimulation of presynaptic ChR2-positive SPNs produced IPSCs in patched spiny neurons similar to earlier studies (Chuhma et al., 2011). Recordings from presynaptic ChR2-positive SPNs showed that APs were reliably evoked by optical stimulation over the time course of the experiments. Application of TTX showed that those responses were mediated by somatic APs, or possibly also ChR2-positive cut axons, and not by direct excitation of ChR2 in the presynaptic terminals. Stimulation of 5-HT1B receptors had no effect on the reliability or half-width of optically evoked APs in the presynaptic neuron. Therefore, the effects of 5-HT1B receptor stimulation were not because of effects on the soma of the presynaptic neuron.

Several pieces of evidence indicate that the locus of action of the 5-HT1B receptor agonist in the present study is the presynaptic terminals of the optically stimulation SPNs, and not the soma of the ChR2-positive neurons. During the period of recording, decay or significant change of AP properties was not observed. Importantly for the interpretation of drug actions, activation of serotonin receptor 5-HT1B with CP-93129 did not impair AP firing or characteristics of ChR2-positive SPNs. This is consistent with previous work showing the 5-HT1B receptor regulates release of neurotransmitters throughout the brain by activating a range of signaling cascades in the presynaptic terminals (Hoyer et al., 2002; Sari, 2004; Masson et al., 2012). Thus, the actions of the 5-HT1B receptor in the present experiments are probably restricted to the axon terminals where the receptor is located. It is possible that 5-HT1B receptors in SPNs only regulate neurotransmitter release and have no additional roles in neuronal network dynamics.

On the cellular level, the distribution of 5-HT1B receptors in presynaptic terminals observed in the present study was consistent with the location predicted from the literature (Boschert et al., 1994; Ghavami et al., 1999; Hoyer et al., 2002; Sari, 2004). The signal was scattered throughout the neuropil in small puncta suggestive of receptor concentrated in presynaptic terminals and varicosities (Ghavami et al., 1999; Di Matteo et al., 2008). The presence of puncta around cell bodies could be interpreted as indicating multiple 5-HT1B-positive synapses on somata. Consistent with this, the collaterals of SPNs form synaptic contacts on the soma of other SPNs (Wilson and Groves, 1980; Bolam and Izzo, 1988; Oorschot et al., 2013) and also on the soma of CINs (Lee et al., 1997). This presynaptic location of 5-HT1B receptors suggests that serotonin is capable of modulating lateral inhibition by SPNs.

The effects of stimulating 5-HT1B receptors on IPSCs suggest that the reduction of the average amplitude is caused in part by an increase in the failure rate of synaptic transmission. The present findings, however, cannot exclude the possibility of an effect on quantal size, because the optically evoked responses may not be unitary IPSCs. While an increase in failure rate does not exclude a change in quantal size, it is consistent with previous work showing that presynaptic serotonin 5-HT1B receptors modulate release of GABA from the terminals of SPNs in their target nuclei, the GP and SNr (Johnson et al., 1992; Stanford and Lacey, 1996; Hoyer et al., 2002; Sari, 2004).

In contrast to previously reported effects of dopamine receptors on lateral inhibition, which differed according to the subtype of presynaptic neuron (Tecuapetla et al., 2009; Dobbs et al., 2016), in the present experiments, the degree of reduction of lateral inhibition caused by 5-HT1B receptor activation was similar for connections of D1 and D2-SPNs. This suggests that the regulatory effect of 5-HT1B is consistent across the different connections between subtypes of SPNs. The effect of 5-HT1B on SPNs of the same subtype was not investigated in the present study, because neurons of the same subtype would express ChR2, so that the direct effects of optical stimulation would have confounded measures of synaptic efficacy.

Despite the effects of 5-HT1B receptor stimulation on collaterals of SPNs, the present research found no evidence to suggest that inhibition by FSIs was modulated by 5-HT1B receptors. IPSCs that occurred in the period 30–100 ms after optical stimulation were not modulated by 5-HT1B receptor stimulation. The events recorded in this period probably include spontaneous IPSCs from FSIs. The IPSCs recorded in any period include a mixture of evoked and spontaneous IPSCs. In the initial 10-ms period after stimulation, evoked IPSCs are expected to be much more numerous than spontaneous IPSCs that happen to occur in that interval. The interval between 10 and 30 ms probably contains a mixture of longer-latency evoked responses and spontaneous IPSCs. For example, IPSCs with a latency of up to 20 ms have been reported in dual patch-clamp recordings (Taverna et al., 2008). However, IPSCs occurring in the interval 30–100 ms after optical stimulation are sure to be spontaneous, because latencies of >30 ms are not credible. The contribution of different cell types to these late interval IPSCs is unknown, but it probably includes both SPN and FSI sources, as well as other GABA interneurons. Our finding that 5-HT1B receptor stimulation did not reduce IPSCs evoked by optogenetic stimulation of FSIs supports the hypothesis that the IPSCs that occurred in the period 30–100 ms (which were not modulated by 5-HT1B receptor stimulation) included spontaneous IPSCs from FSIs.

Lateral inhibition between SPNs is also strongly modulated by dopamine. While serotonin seems to trigger a similar IPSC reduction in both SPN subtypes, dopamine interaction is more complex. Several studies showed strong dopaminergic inhibition of D2→D1/D2 connections, mediated by presynaptic D2 receptors (Tecuapetla et al., 2009; Kohnomi et al., 2012; Dobbs et al., 2016). Dopamine D1 receptors have been shown to both selectively inhibit (Hjelmstad, 2004; Kohnomi et al., 2017) and facilitate lateral inhibition (Wei et al., 2017) but have no effect in the dopamine depleted striatum (Wei et al., 2017).

In addition, Mathur and colleagues showed that presynaptic 5-HT1B receptor activation caused long-term depression of corticostriatal synapses (Mathur et al., 2011). This suggests that 5-HT1B receptors suppress network level activity and output of the striatum by reducing excitatory input. In contrast, our findings show disinhibition between SPNs after 5-HT1B receptor activation, by reducing lateral inhibition, which would promote overall striatal activity and output. However, these actions are not contradictory. Long-term depression of corticostriatal synapses would continue longer than the direct effects on lateral inhibition that we observed. Serotonin 5-HT1B receptor-mediated long-term depression of corticostriatal synapses might act as a high pass filter for strong cortical input (Mathur and Lovinger, 2012), in which case the transient disinhibitory effect we described would amplify the effect of the strongest excitatory inputs.

The functional implications of decreasing lateral inhibition in the striatum are yet to be determined. Speculatively, reducing GABA release in the striatum would disinhibit SPNs and lower the contrast between SPNs, making different ensembles of active SPNs less separated. This may eventually promote more random/flexible striatal outcomes from excitatory input by making it easier to switch between less-defined synchronized clusters encoding certain behaviors (Burke et al., 2017). Concurrently, in the SNr and GP, 5-HT1B receptor activation reduces inhibition from spiny neurons, lowering the overall output from the striatum. By reducing the striatal contrast and decreasing basal ganglia output, serotonin acting via 5-HT1B receptors on SPNs might lower the threshold for striatal action selection and in turn promote more flexible switching between different behaviors.
